# GADD34 Keeps the mTOR Pathway Inactivated in Endoplasmic Reticulum Stress Related Autophagy

**DOI:** 10.1371/journal.pone.0168359

**Published:** 2016-12-16

**Authors:** Marianna Holczer, Gábor Bánhegyi, Orsolya Kapuy

**Affiliations:** Department of Medical Chemistry, Molecular Biology and Pathobiochemistry, Semmelweis University, Budapest, Hungary; University of Hong Kong, HONG KONG

## Abstract

The balance of protein synthesis and proteolysis (*i*.*e*. proteostasis) is maintained by a complex regulatory network in which mTOR (mechanistic target of rapamycin serine/threonine kinase) pathway and unfolded protein response are prominent positive and negative actors. The interplay between the two systems has been revealed; however the mechanistic details of this crosstalk are largely unknown. The aim of the present study was to investigate the elements of crosstalk during endoplasmic reticulum stress and to verify the key role of GADD34 in the connection with the mTOR pathway. Here, we demonstrate that a transient activation of autophagy is present in endoplasmic reticulum stress provoked by thapsigargin or tunicamycin, which is turned into apoptotic cell death. The transient phase can be characterized by the elevation of the autophagic marker LC3II/I, by mTOR inactivation, AMP-activated protein kinase activation and increased GADD34 level. The switch from autophagy to apoptosis is accompanied with the appearance of apoptotic markers, mTOR reactivation, AMP-activated protein kinase inactivation and a decrease in GADD34. Inhibition of autophagy by 3-methyladenine shortens the transient phase, while inhibition of mTOR by rapamycin or resveratrol prolongs it. Inhibition of GADD34 by guanabenz or transfection of the cells with siGADD34 results in down-regulation of autophagy-dependent survival and a quick activation of mTOR, followed by apoptotic cell death. The negative effect of GADD34 inhibition is diminished when guanabenz or siGADD34 treatment is combined with rapamycin or resveratrol addition. These data confirm that GADD34 constitutes a mechanistic link between endoplasmic reticulum stress and mTOR inactivation, therefore promotes cell survival during endoplasmic reticulum stress.

## Introduction

Choosing between life and death is one of the most important tasks of cells building up an organism. Adaptation to altered environmental conditions by re-modelling its own cell physiology leads to a new homeostatic state, while the failure of adaptation finally results in cell death. Endoplasmic reticulum (ER), a main integrator of signals from the external and internal environment, has key functions in synthesising and packaging secreted and membrane proteins, metabolism (such as lipid biosynthesis and carbohydrate metabolism) and several signalling processes. For these integrated roles of ER a special luminal homeostasis is required; its imbalance might result in ER stress [1–3]. ER stress transiently activates a pro-survival mechanism characterized by a negative balance of proteostasis (i.e. decreased translation and increased proteolysis by ERAD and autophagy), while severe stress leads to apoptotic cell death. It has been reported that ER stress dependent apoptosis is typically preceded by autophagy-dependent self-protection [4]. ER stress dependent processes are regulated by a coordinated systems-level crosstalk between the complex regulatory networks of two signalling pathways [5], the unfolded protein response (UPR) [6] and the mTOR pathway [7].

UPR has three well-defined transducers activated by ER stress, called PERK (PKR-like ER kinase), IRE1 (inositol requiring 1) and ATF6 (activating transcription factor 6) [6]. All three components are ER-resident transmembrane proteins, which become active upon ER stress. While the activation of both IRE1 and ATF6 promotes transcription of UPR target genes (such as chaperones), PERK-controlled pathway leads to the general inhibition of protein translation [6]. The active PERK is able to phosphorylate the translation initiation factor eiF2α to reduce the global protein synthesis and decrease the flux of proteins entering the ER [8]. Interestingly this phosphorylation of eiF2α enhances a transcription activator, so called ATF4 [9]. Two downstream targets of ATF4 are GADD34 (growth arrest and DNA damage-inducible 34) [10] and CHOP (transcription factor C/EBP homologues protein) [11], respectively. GADD34 is a regulatory subunit of PP1 phosphatase [12], while CHOP is a transcription factor that controls gene transcription involved in apoptosis [13]. GADD34 is not only activated by ATF4, but its transcription is also promoted by CHOP-dependent manner with respect to ER stress [14, 15]. Although PERK stays active during ER stress, the level of phosphorylated eiF2α decreases due to GADD34-dependent dephosphorylation [10, 15]. Some experimental data suggest that this dephosphorylation of eiF2α has a crucial protective feature by enhancing adaptation to ER stress [15]. ER stress stimuli induced protein expressions (such as ATF4 and CHOP) were drastically reduced in GADD34-/- mouse embryonic fibroblasts [16]. Accordingly, both GADD34 overexpression and catalytically inactive GADD34 addition (GADD34ΔC/ΔC) result in premature cell death in the presence of ER stress [11, 17], but the detailed mechanism is still a mystery.

FRAP/mTOR is the key Ser/Thr protein kinase of mTORC1 complex and is the main component of mTOR pathway [7, 18]. The complex is the master regulator of cellular homeostasis by integrating different external and internal signals, such as growth factors, amino acids, glucose and energy status [19]. mTORC1 gets activated by a phosphatidylinositol 3 kinase (PI3K)—Akt kinase-dependent manner *via* TSC1/TSC2 [7]. Beside mTOR, AMPK (AMP activated protein kinase) also senses cellular energy status and maintains energy homeostasis. AMPK directly inhibits mTORC1 complex via phosphorylation, meanwhile the kinase is able to induce autophagy by phosphorylating ULK-1, one of the key activators of self-cannibalism during nutrient depletion [20]. Inhibition of mTORC1 by nutrient depletion or rapamycin addition results in autophagy and in a block of protein translation by dephosphorylating the crucial targets of mTOR, such as ribosomal protein S6 kinase (p70S6) and translation initiation factor 4E binding protein-1 (4-EBP1) [7, 19]. Novel findings have revealed that the mTOR-AMPK crosstalk is highly regulated by resveratrol, a naturally occurring polyphenol found in grapes. Resveratrol addition seems to be a promising treatment for various diseases with mTOR hyperactivation (such as cancer, lymphangioleiomyomatosis). Resveratrol is able to down-regulate mTORC1 both directly and indirectly via AMPK activation, therefore it promotes autophagy [21, 22].

Although the cellular roles of UPR and mTOR pathways seem to be different, an intensive crosstalk between the two mechanisms has been revealed with respect to cellular stress [23]. The down-regulation of mTOR by rapamycin enhances cell viability during UPR-induced ER stress showing an existence of a crosstalk between UPR and mTOR pathways [24]. The activation of mTOR is drastically down-regulated at excessive level of ER stress, while mTOR inhibition increases cell viability via autophagy induction. It was shown that excessive ER stress is coupled to chronic activation of mTOR resulting in the downstream activation of both PERK and IRE1 branches of UPR and in apoptotic cell death [25, 26]. Pharmacological induction of UPR activates mTOR, therefore inhibits autophagy [25, 27]. Constitutive mTOR activation by loss of TSC1/TSC2 rapidly stimulates the targets of both PERK and IRE1, which UPR activation could not be observed in combined treatment of TSC1/TSC2 depletion and rapamycin addition. These results confirm that mTOR contributes to ER-stress-induced self-killing mechanism [26, 28].

Beside ER stress-dependent regulation of GADD34, it is also induced by DNA damage and viral infection. The tumor suppressor Drs was able to down-regulate viral replication via complex formation with GADD34/TSC1/2. The interaction of Drs with GADD34/TSC1/2 results in suppression of mTOR pathway, while mTOR targets remain phosphorylated in drs-KO MEFs [29]. The viral protein of human T-cell leukemia virus type-1 (HBZ) also promotes the activation of mTOR pathway throughout GADD34 inhibition. HBZ negatively controls the cellular localization of GADD34 with direct interaction [30]. Starvation-induced autophagy is significantly down-regulated by overexpression of HBZ, meanwhile the important cytoprotective role of GADD34-induced autophagy during starvation was also proved [31]. GADD34 knock out mice have highly phosphorylated mTOR pathway at nutrient depletion suggesting that GADD34 negatively regulates mTOR by dephosphorylating TSC2 [31]. GADD34 also induced cytoprotective autophagy by down-regulating mTOR pathway via TSC2-dependent dephosphorylation in mutant huntingtin expressing cells. GADD34-controlled transient dephosphorylation of mTOR targets was observed when mutant huntingtin fragment proteins were added to PC6.3 cells [32]. It was also shown that GADD34 was able to postpone apoptosis via enhancement of autophagy and down-regulation of mTOR by LPS stimulus in macrophages [33]. These results suggest that GADD34 has a crucial role in down-regulating mTOR pathway at various cellular stress events.

The aim of the present study was to verify the mechanistic role of GADD34 in connecting mTOR pathway to ER stress. By using pharmacological tools and GADD34 silencing we defined a crucial interaction between UPR-induced GADD34 and mTOR pathway during ER stress. In particular, we found that GADD34 level got transiently increased with excessive level of ER stress. This phenotype was highly correlated to a transient mTOR inactivation and autophagy induction. However the turning on of apoptosis immediately re-activated mTOR, meanwhile GADD34 got diminished suggesting an important role of GADD34 in autophagy-dependent survival. With GADD34 depletion we identified GADD34-dependent crucial effect on mTOR pathway during ER stress. This study also suggested that resveratrol treatment might be able to compensate the negative effect of GADD34 silencing with respect to ER stress.

## Materials and Methods

### Materials

Thapsigargin (Sigma-Aldrich, T9033), rapamycin (Sigma-Aldrich, R0395), tunicamycin (Sigma-Aldrich, T7765) and 3-methyladenine (Sigma-Aldrich, M9281), resveratrol (Sigma-Aldrich, R5010), guanabenz (Sigma-Aldrich, G110), Bafilomycin A (Sigma-Aldrich, M17931) were purchased. All other chemicals were of reagent grade.

### Cell culture and maintenance

As model system, human embryonic kidney (HEK293T, ATCC, CRL-3216) and human liver carcinoma (HepG2, ATCC, HB-8065) cell lines were used. It was maintained in DMEM (Life Technologies, 41965039) medium supplemented with 10% fetal bovine serum (Life Technologies, 10500064) and 1% antibiotics/antimycotics (Life Technologies, 15240062). Culture dishes and cell treatment plates were kept in a humidified incubator at 37°C in 95% air and 5% CO_2_.

### SDS-PAGE and Western blot analysis

Cells were harvested and lysed with 20 mM Tris, 135 mM NaCl, 10% glycerol, 1% NP40, pH 6.8. Protein content of cell lysates was measured using Pierce BCA Protein Assay (Thermo Scientific, 23225). During each procedure equal amounts of protein were used. SDS-PAGE was done by using Hoefer miniVE (Amersham). Proteins were transferred onto Millipore 0.45 μM PVDF membrane. Immunoblotting was performed using TBS Tween (0.1%), containing 5% non-fat dry milk for blocking membrane and for antibody solutions. Loading was controlled by developing membranes for GAPDH or dyed with Ponceau S in each experiment. For each experiment at least three independent measurements were carried out. The following antibodies were applied: antiLC3B (SantaCruz, sc-16755), anticaspase-3 (SantaCruz, sc-7272), antiPARP (Cell Signaling, 9542S), antiULK555P (Cell Signaling, 5869S), antiULK (Cell Signaling, 8054S), antip70S6P (Cell Signaling, 9234S), antip70S6 (SantaCruz, sc-9202), anti4EBP1P (Cell Signaling, 9459S), anti4EBP1 (Cell Signaling, 9644S), antiGADD34 (SantaCruz, sc-8327), antieiF2αP (SantaCruz, sc-9721L), antieiF2α (SantaCruz, sc-9722S), antip62 (Cell Signaling, 5114S) and antiGAPDH (Santa Cruz, 6C5), HRP conjugated secondary antibodies (SantaCruz, sc-2020 and Cell Signaling, 7074S, 7076S).

### RNA interference

RNA interference experiments were performed using Lipofectamine RNAi Max (Invitrogen) in GIBCO^™^ Opti-MEM I (GlutaMAX^™^-I) Reduced-Serum Medium liquid (Invitrogen) and 20 pmol/ml siRNA. The siGADD34 oligonucleotides were purchased from ThermoFisher (HSS177543). 200 000 HEK293T cells were incubated at 37°C in a CO_2_ incubator in antiobiotic free medium for 16 hours, then the RNAi duplex-Lipofectamine^™^ RNAiMAX complexes were added to the cells for overnight. Then fresh medium was added to the cells and the appropriate treatment was carried out.

### RNA extraction and real-time PCR

Total RNA content of cells was extracted using TRIzol RNA isolation reagent (Invitrogen) [34]. Retrotranscription was performed using SuperScriptII First-Strand Synthesis System (Invitrogen). Nucleic acid levels were measured using GenQuant pro RNA/DNA calculator. Equal amounts of cDNA were used for real-time PCR to check the efficiency of Gadd34 silencing. PCR reaction and real-time detection was performed using GoTaq(R) qPCR Master Mix (Promega, A6002) and STRATAGENE Mx3005P Real-Time PCR Detection System. The real-time PCR thermocycles were the followings: 95°C 10 min (1x), 95°C 30 sec, 58°C 45 sec, 72°C 30 sec, (40x), 95°C 5 min, 55°C 1 min, 97°C 30 sec (1x). The appropriate forward and reverse real-time PCR primers were used for Gadd34 and GAPDH.

### Cell viability assays

The relative amount of viable cells was calculated by Burker chambers. Cell viability was detected using CellTiter-Blue assay (Promega, G8080). Cells were grown and treated on 96-well plates, and were incubated with resazurin for 2 h at 37°C. Absorbance was measured at 620 nm, and expressed in arbitrary unit, being proportional to cell toxicity. For each of these experiments at least three parallel measurements were carried out.

### Statistics

For densitometry analysis Western blot data were acquired using ImageQuant 5.2 software. The relative band densities were shown and normalized to an appropriate total protein or GAPDH band used as reference protein (see Supplementary Information). For each of the experiments three independent measurements were carried out. Results are presented as mean values ± S.D. and were compared using ANOVA with Tukey’s multiple comparison post hoc test. Asterisks indicate statistically significant difference from the appropriate control: ∗—p < 0.05; ∗∗—p < 0.01.

## Results

### mTOR pathway becomes transiently down-regulated during excessive level of ER stress

**Fig 1:**
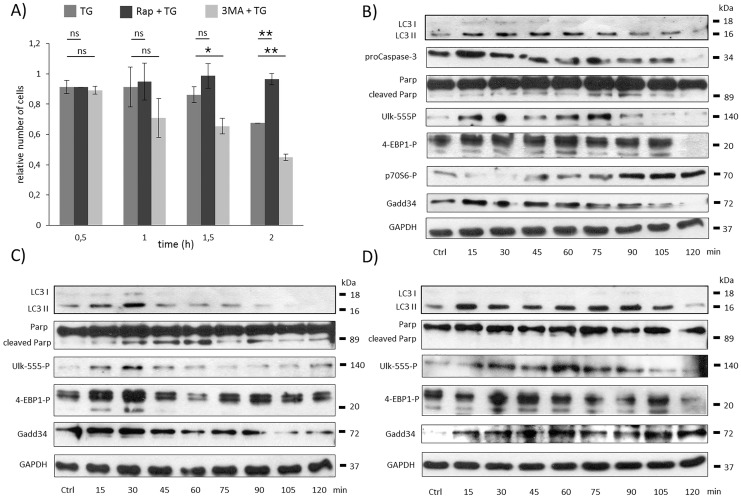
Time course profile of cell viability, autophagy and apoptosis in TG-induced ER stress with/without addition of autophagy activator/inhibitor. **A)** HEK293T cells were treated with 10 μM TG for two hours and pre-treated with rapamycin (100 nM for two hours) or 3-MA (1 mM for two hours) followed by TG addition (10 μM for two hours), meanwhile the relative number of viable cells was denoted in time (error bars represent standard deviation, asterisks indicate statistically significant difference from the control: ∗—p < 0.05; ∗∗—p < 0.01). **B)** During TG treatment the markers of autophagy (LC3), apoptosis (proCaspase3, PARP), AMPK activation (ULK-555P) and mTOR activation (4-EBP1P, p70SP), as well as GADD34 were followed in time by immunoblotting. During pre-treatmetn with **C)** 3-MA and **D)** rapamycin followed by TG addition the autophagy (LC3), the apoptosis (PARP), the AMPK (ULK-555P) and the mTOR (4-EBP1P) markers and GADD34 were followed in time by immunoblotting. GAPDH was used as loading control.

We previously confirmed by using both theoretical and experimental techniques that ER stress induced apoptotic cell death is always preceded by autophagy-dependent survival. Both mild and excessive levels of ER stress induce autophagy both in HepG2 and HEK293T cells, however the self-cannibalism has only a transient activation at high level of ER stress followed by a switch-like activation of apoptotic cell death [4]. The dynamical characteristic of autophagy-apoptosis crosstalk was described by a double negative feedback loop between the autophagy and apoptosis inducers [35]. However new results suggested that mTOR pathway might also play an essential role in the ER stress response mechanism. To further confirm the presence of UPR-mTOR crosstalk HEK293T cells were treated with a high dose of thapsigargin (TG– 10 μM), a commonly used drug to perturb ER homeostasis. First both the relative cell number and relative cell viability were followed in time (Fig 1A and S1A Fig). The results suggest that cells remained alive for a one-and-a-half hour-long treatment, but their viability quickly dropped after two hours.

To detect the activation profile or level of the key molecules of autophagy (such as LC3I and II), apoptosis (proCaspase-3, PARP), AMPK and mTOR pathway during ER stress immunoblotting were performed (Fig 1B and S1B Fig). Transient activation of autophagy was observed between 15 and 75 min. Later the amount of LC3II/I decreased drastically; meanwhile the activation of apoptosis was indicated by intensive decrease in proCaspase-3 level and by PARP cleavage. These data confirm our previous results, namely autophagy starts to compensate the negative effects of ER stressor, but finally apoptosis kills the cells during excessive level of ER stress.

The activity of AMPK and mTOR showed a transient, biphasic antiparallel profile with respect to ER stress (Fig 1B and S1B Fig). The activation of AMPK was detected between 15 and 75 min by the phosphorylation on Ser-555 residue of ULK-1, one of the key promoters of autophagy. Meanwhile the inactivation of mTOR was observed, followed by the dephosphorylation of p70S6 and phosphorylation of lower band of 4-EBP1, respectively (Fig 1B and S1B Fig). Parallel to the activation of apoptotic markers after 75 min long treatment, mTOR quickly got reactivated supposing that mTOR pathway has some role in the self-killing mechanism. Remarkably, mTOR became even more active, than it was previously detected under physiological conditions. On the other hand, AMPK activity completely disappeared after 90 min-long TG treatment. These results suggest that mTOR pathway has to be transiently down-regulated and AMPK has to be transiently up-regulated corresponding to the temporary activation of autophagy followed by apoptosis with respect to excessive level of ER stress.

To confirm that the dynamic characteristic of both AMPK and mTOR is connected to autophagy-apoptosis crosstalk during ER stress cells were pre-treated with autophagy inhibitor (3-methyladenine (3-MA)– 1 mM for 2 hours) or autophagy activator/mTOR inhibitor (rapamycin (Rap)– 100 nM for 2 hours) followed by 2 hour-long TG (10 μM) treatment. The relative amount of viable cells are drastically dropped after 1.5 h of TG addition when cells were pre-treated with 3-MA suggesting that autophagy-dependent survival was successfully blocked (Fig 1A and S2A Fig). Immunoblot data suggest that autophagy got activated for a short period only, since LC3II/I level was observed after 30 min of TG addition meanwhile apoptosis quickly got active (Fig 1C and S2B Fig). Although the regulatory system tried to promote AMPK activation and mTOR inactivation (see ULK-555 and 4-EBP-1 phosphorylations between 15–30 min on Fig 1C and S2B Fig), apoptosis quickly got activated coincided with AMPK inactivation and intensive mTOR re-activation, respectively. These data confirm that mTOR pathway might have a crucial contribution in apoptosis during ER stress.

However, the pre-treatment with rapamycin was able to extend autophagy-dependent cell viability and delay apoptotic cell death during ER stress (Fig 1A and S3 Fig). According to the high level of LC3II/I, autophagy remained active even 105 min after TG addition, meanwhile the apoptosis marker was inactive, namely no PARP cleavage was observed (Fig 1D and S3B Fig). The ULK-555 phosphorylation referred to an active AMPK, meanwhile mTOR remained completely inhibited (see 4-EBP-1 phosphorylation between 15–105 min on Fig 1D and S3B Fig). These data suggest that the precise AMPK-mTOR balance is highly connected to autophagy-apoptosis crosstalk in respond to ER stress.

To test whether autophagy is functioning properly in the above mentioned experiments the treatments were repeated in the presence of Bafilomycin A (Baf). Baf is a well-known inhibitor of autophagic flux by preventing the fusion between autophagosomes and lysosomes [36]. 2 hours long pre-treatment with 100 nM Baf clearly increased the level of LC3II/I in all the three cases (TG, Rap+TG, 3MA+TG treatments, respectively) (S4 Fig). To further confirm the autophagic mechanism p62 was also detected. The drastic decrease of p62 level was completely diminished in the presence of Baf supposing a complete block of autophagic flux (S4 Fig). Although the width of the autophagic window can be different, these results suggest that a transient activity of autophagy is always observed with respect to ER stress.

Similar effects were observed by using tunicamycin, another well-known ER stressor (data not shown).

### GADD34 gets transiently activated parallel to autophagy induction during ER stress

The increasing level of UPR-induced GADD34 (a regulatory subunit of PP1) was already observed with respect to ER stress [37]. To identify the role of GADD34 during TG-activated ER stress its protein level was detected by immunoblotting (Fig 1B and S1B Fig). Our results showed a transient increase of GADD34 level between 15 and 75 min of TG treatment, however its level got completely diminished later. This increase in GADD34 level was highly correlated to the autophagy/AMPK activation and mTOR inactivation; however GADD34 quickly disappeared from the cell when mTOR got re-activated and apoptotic cell death turned on.

TG addition to cells pre-treated with 3-MA resulted in an early drop of GADD34 protein level, meanwhile rapamycin pre-treatment followed by TG was able to maintain GADD34 level even after two hours of ER stressor addition (Fig 1C and 1D, S2B and S3B Figs). These results suggest that GADD34 might be related to the life-and-death-decision controlled by autophagy-apoptosis crosstalk during ER stress. Previous data have shown that GADD34 has a negative effect on mTOR pathway via TSC2 activation at various stress events [31, 32]. Our data suggest that GADD34 might have a role in promoting autophagy-dependent survival by down-regulating mTOR pathway with respect to excessive level of ER stress.

### Inhibition of GADD34 reduces cell viability via mTOR-dependent hyper-activation

**Fig 2:**
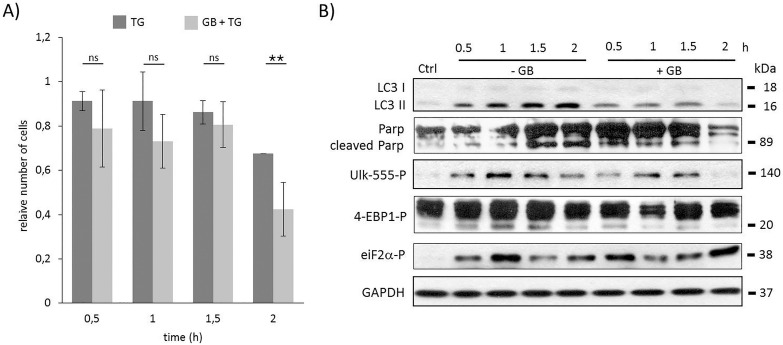
Time course profile of cell viability, autophagy and apoptosis in TG-induced ER stress when GADD34 was inhibited. HEK293T cells were pre-treated with GB (5 μM for one hour) followed by TG addition (10 μM for two hours). The GB level was kept high until end of the cell treatment. **A)** The relative number of viable cells after TG treatment was denoted in time (error bars represent standard deviation, asterisks indicate statistically significant difference from the control: ∗—p < 0.05; ∗∗—p < 0.01). **B)** Markers for autophagy (LC3), apoptosis (PARP), AMPK activation (ULK-555P) and mTOR activation (4-EBP1P) as well as eiF2αP were followed in time by immunoblotting. GAPDH was used as loading control.

Since GADD34 protein level has shown an interesting transient time course profile with respect to ER stress, we were interested in whether GADD34 inhibition is able to affect the response mechanism. For GADD34 inhibition its well-known pharmacological inhibitor, guanabenz (GB) was used. GB binds selectively to the regulatory subunit protein phosphatase 1, therefore disrupting the stress-induced dephosphorylation effect of GADD34 [38]. In order to choose the proper concentration for GB, cell viability assay was carried out with different concentrations of the inhibitor (S5 Fig). The one hour-long treatment with GB at low concentration (0.01 μM) already decreased cell viability, suggesting that GADD34 might have a role in protecting the cells. When HEK293T cells pre-treated with GB for one hour (5 μM) were treated with TG (10 μM), a drastic decrease was observed in the amount of viable cells (Fig 2A and S6A Fig). This negative effect of GB was even more severe after two hours long TG treatment supposing that GADD34 inhibition might be able to speed up cell death process with respect to severe ER stress.

To explore the role of GADD34 during ER stress the essential markers of autophagy, apoptosis, mTOR and AMPK pathways were followed in time (Fig 2B and S6B Fig). GB does not affect the level of GADD34; rather it blocks its catalytic activity [38]. Therefore to check whether the treatment was effective, the phosphorylation level of eIF2α, the main ER stress-induced GADD34 target, was also followed by immunoblotting (Fig 2B and S6B Fig). Since the level of phosphorylated eIF2α was not diminished, we could assume that GB completely blocked GADD34 activity even after two hours long TG treatment. Comparing the time course data of this combined treatment (GB+TG) with “simple” TG treatment the intensity of LC3II/Iwas much weaker after one hour of TG addition suggesting that the autophagy-dependent survival was down-regulated in the absence of GADD34. In addition, PARP cleavage was already detected at half hour long combined treatment (GB+TG) assuming that the fast inhibition of autophagy let the apoptotic process to be active. The turning on of apoptosis was correlated to the decrease of both relative cell viability and cell number (Fig 2A and S6A Fig). According to the time profile of autophagy AMPK seemed to be down-regulated, meanwhile mTOR was up-regulated when GADD34 was inhibited by GB during TG treatment. The relative amount of phosphorylated Ulk-555 was much lower; however the weak phosphorylation of lower band of 4-EBP1 suggested that mTOR became active already after half hour long TG treatment in the absence of active GADD34. These results suppose that the presence of GADD34 might be able to postpone apoptotic cell death via mTOR inhibition/AMPK up-regulation with respect to excessive level of ER stress.

Similar effects of GADD34 were observed by using another human cell line (HepG2) and another well-known ER stressor (tunicamycin) (see S7 and S8 Figs). These results suggest that the answer mechanism might accomplish a universal characteristic with respect to ER stress.

### Inactivation of mTOR can compensate for GADD34 inhibition with respect to ER stress

**Fig 3:**
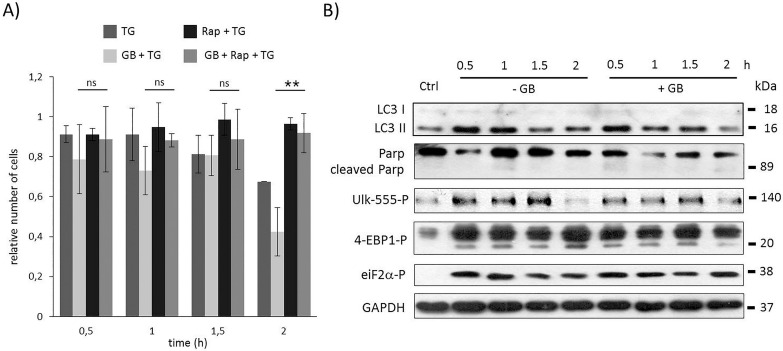
Time course profile of cell viability, autophagy and apoptosis in TG-induced ER stress when both GADD34 and mTOR was inhibited. HEK293T cells were pre-treated with GB (5 μM for one hour) then with rapamycin (100 nM for two hours) followed by TG addition (10 μM for two hours). The GB level was kept high until end of the cell treatment. **A)** The relative number of viable cells after TG treatment was denoted in time (error bars represent standard deviation, asterisks indicate statistically significant difference from the control: ∗—p < 0.05; ∗∗—p < 0.01). **B)** The autophagy (LC3), the apoptosis (PARP), the AMPK (ULK-555P) and the mTOR (4-EBP1P) markers and eiF2αP were followed in time by immunoblotting. GAPDH was used as loading control.

To explore whether GADD34 is able to help autophagy *via* mTOR down-regulation, the GADD34 inhibition was combined with rapamycin treatment during ER stress. TG-treated HEK293T cells were pre-treated first with GB (5 μM– 1 hour) then with rapamycin (100 nM– 2 hours), meanwhile GB level was maintained until at the end of the treatment with the ER stressor (TG, 10 μM– 2 hours). Although GADD34 inhibition drastically decreased the amount of viable cells, blocking mTOR pathway by rapamycin could diminish its negative effect suggesting that GADD34 has an important role in mTOR inhibition in response to ER stress (Fig 3A and S9A Fig). Similarly to rapamycin pre-treatment combined treatment with GB and rapamycin during ER stress resulted in an intensive autophagy process, namely its activity was maintained even after two hours long TG treatment (see high LC3II/I level on Fig 3B and S9B Fig), meanwhile apoptotic cell death was delayed (see the absence of PARP cleavage on Fig 3B and S9B Fig). The appearance of lower phosphorylation band of 4-EBP1 suggested that mTOR was inactive; while the intensive phosphorylation of ULK-555 referred to AMPK activity. Our experimental data confirm that UPR-induced GADD34 has an essential role in delaying apoptotic cell death via mTOR inhibition/AMPK up-regulation in ER stress.

### Resveratrol-dependent mTOR inactivation can rescue GADD34 inhibition with respect to ER stress

**Fig 4:**
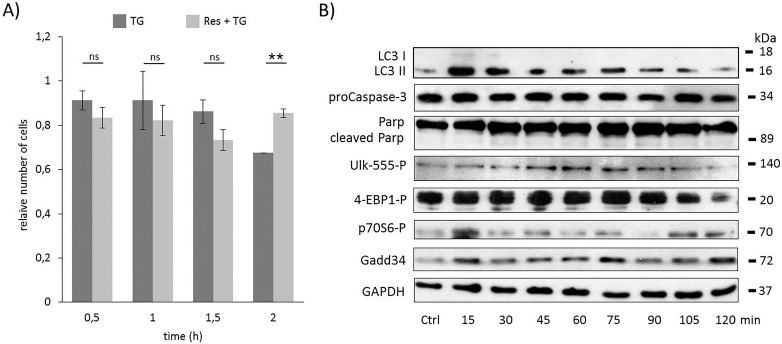
Time course profile of cell viability, autophagy and apoptosis in TG-induced ER stress when autophagy was hyper-activated by resveratrol. HEK293T cells were pre-treated with resveratrol (10 μM for twenty-four hours) followed by TG addition (10 μM for two hours). **A)** The relative number of viable cells after TG treatment was denoted in time (error bars represent standard deviation, asterisks indicate statistically significant difference from the control: ∗—p < 0.05; ∗∗—p < 0.01). **B)** The relative cell viability was plotted in time after TG treatment (error bars represent standard deviation). **C)** The autophagy (LC3), the apoptosis (proCaspase3, PARP), the AMPK (ULK-555P) and the mTOR (4-EBP1P, p70SP) markers and GADD34 were followed in time by immunoblotting. GAPDH was used as loading control.

As it was mentioned before resveratrol is able to down-regulate mTOR and up-regulate AMPK, therefore induces autophagy [22]. To confirm its positive role during ER stress cells were pre-treated with resveratrol (10 μM) for 24 hours then ER stress was induced by TG (10 μM– 2 hours). The experimental data of both the relative cell number and cell viability suggest that resveratrol pre-treatment similarly to rapamycin was able to extend cell viability and postpone cell death (Fig 4A and S10A Fig). To verify this effect of resveratrol the key molecules were also followed in time by immunoblotting (Fig 4B and S10B Fig). The intensive presence of LC3II/Isupposed that autophagy remained active even after two hours long TG treatment, however neither pro-Caspase3 depletion nor PARP cleavage were detected. These results suggest that resveratrol like rapamycin is able to delay apoptotic cell death through intensive autophagy-dependent survival with respect to ER stress.

The phosphorylations of both ULK on its Ser-555 residue and the lower band of 4-EBP1 show that AMPK was active, meanwhile mTOR pathway got inactive until end of the combined treatment (Fig 4B and S10B Fig). These results confirm that resveratrol induces autophagy *via* disrupting the mTOR-AMPK balance. The UPR-induced GADD34 protein level was also kept high, confirming its important role in autophagy-dependent survival during ER stress.

**Fig 5:**
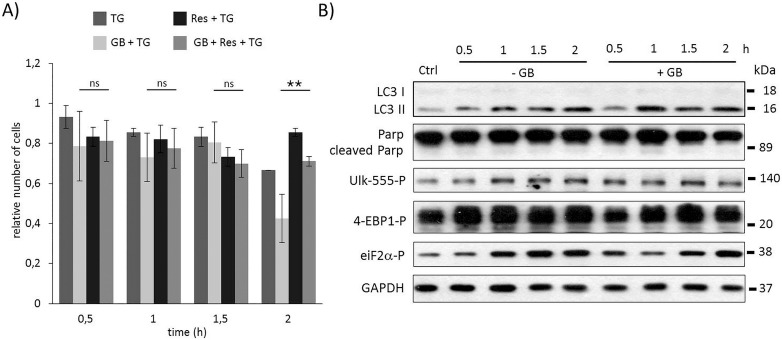
Time course profile of cell viability, autophagy and apoptosis in TG-induced ER stress when GADD34 inhibition is combined with resveratrol addition. HEK293T cells were pre-treated with GB (5 μM for one hours) then with resveratrol (10 μM for twenty-four hours) followed by TG addition (10 μM for two hours). The GB level was kept high until end of the cell treatment. **A)** The relative number of viable cells after TG treatment was represented in time (error bars represent standard deviation, asterisks indicate statistically significant difference from the control: ∗—p < 0.05; ∗∗—p < 0.01). **B)** The autophagy (LC3), the apoptosis (PARP), the AMPK (ULK-555P) and the mTOR (4-EBP1P) markers and eiF2αP were followed in time by immunoblotting. GAPDH was used as loading control.

To test whether resveratrol pre-treatment can rescue GB addition during TG-induced ER stress a special combined treatment was carried out. First cells were treated with GB (5 μM– 1 hour), then resveratrol was added (10 μM) for 24 hours. The level of GB was kept high until end of the treatment, while resveratrol was washed out before ER stress was induced by TG (10 μM– 2 hours). Similarly to the rapamycin, resveratrol pre-treatment was able to extend cell viability in GB pre-treated cells with respect to ER stress (Fig 5A and S11A Fig). Following both the autophagy and apoptosis markers, using Western blot, intensive autophagy (see the LC3II/I on Fig 5B and S11B Fig) was observed until end of the treatment, meanwhile apoptosis induction (absence of PARP cleavage on Fig 5B and S11B Fig) was not detected. These data suggest that cell viability was maintained due to autophagy-dependent survival; although GADD34 was inactivated by GB (the inactivation of GADD34 was detected by eif2alpha-P). In this combined treatment resveratrol was able to hyper-activate AMPK (see ULK-555P on Fig 5B and S11B Fig) and down-regulate mTOR (intensive phosphorylation of low band of 4-EBP1P). These results confirm that the negative effect of blocking GADD34 by GB can be suppressed by mTOR inhibition (AMPK activation) and resveratrol seems to be a promising compound to extend cell viability via autophagy induction with respect to ER stress.

### GADD34 silencing by siRNA has similar effects to GB treatment with respect to ER stress

**Fig 6:**
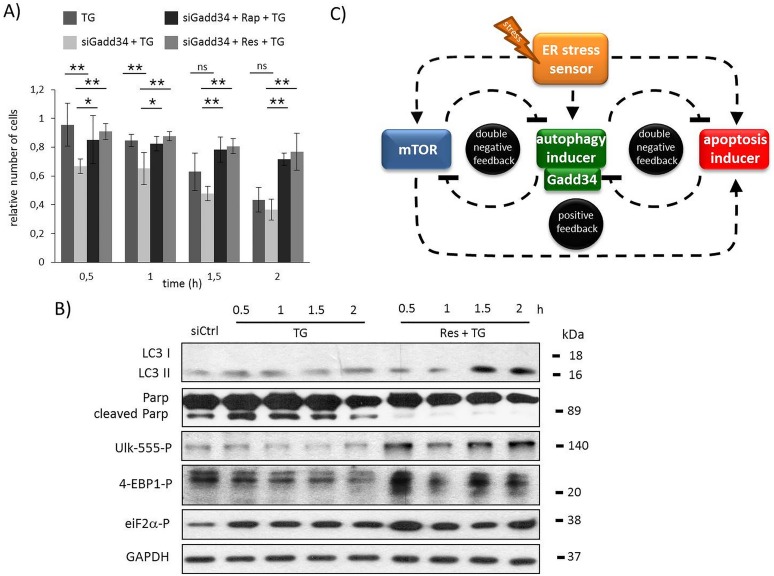
GADD34 down-regulates mTOR pathway during TG-induced ER stress. GADD34 was silenced in HEK293T cells, then cells were treated with 10 μM TG for two hours and pre-treated with resveratrol (10 μM for twenty-four hours) followed by TG addition (10 μM for two hours). **A)** The relative number of viable cells after TG treatment was represented in time (error bars represent standard deviation, asterisks indicate statistically significant difference from the control: ∗—p < 0.05; ∗∗—p < 0.01). **B)** The autophagy (LC3), the apoptosis (PARP), the AMPK (ULK-555P) and the mTOR (4-EBP1P) markers and GADD34 were followed in time by immunoblotting. GAPDH was used as loading control. **C)** The wiring diagram of the control network with respect to ER stress. The mTOR, the autophagy inducer, the apoptosis inducer and the ER stress sensors are denoted by isolated blue, green, red and orange boxes, respectively. Dashed line shows how the components can influence each other, while blocked end lines denote inhibition.

To confirm the essential role of GADD34 in ER stress response mechanism the experiments was done by silencing GADD34 with siRNA (S12A and S12B Fig). Similar to GB addition GADD34 silencing drastically decreased the amount of viable cells during TG treatment, while pre-treatment with resveratrol was able to keep the cells alive (Fig 6A). Using siGADD34 autophagic response is much weaker meanwhile apoptotic cell death turns on half an hour earlier with respect to ER stress (Fig 6B and S12B Fig). On the contrary, resveratrol pre-treatment could extend autophagy even in the absence of GADD34, while apoptotic cell death was delayed (Fig 6B and S12C Fig). In these combined treatments the AMPK maintained its active state (see ULK-555P on Fig 6B and S12C Fig), parallel the mTOR remained inactive (see 4-EBP1P on Fig 6B and S12C Fig). These data further confirm that the negative effect of GADD34 silencing can be rescued by mTOR down-regulation/AMPK up-regulation during ER stress.

## Discussion

The maintenance of intrinsic homeostasis in living organisms is mainly dependent on the ability of cells to take precise actions with respect to various stimuli (such as nutrient availability, inflammatory mediators, growth factors etc.) [39, 40]. The generated response mechanism has to provide an accurate decision to avoid any “misunderstanding” and their fatal consequences. The existence of a crosstalk between two signalling pathways traditionally considered as separate ones (UPR [6] and mTOR pathways [7]) has been highlighted recently [5]. On this basis we have recently proposed a regulatory network, where the life-and-death decision of ER stress response mechanism is defined by the positive and negative feedback loops of autophagy, apoptosis and mTOR pathways (see Fig 5A in [41]). In that paper each promoter of autophagy-dependent survival was called autophagy inducer and the key components of apoptotic cell death was defined as apoptosis inducer, respectively [33]. Although many components of these three pathways are already known, some of them are still missing. In this study we tried to explore the mechanistic connection between mTOR and UPR pathways during ER stress.

First we followed the time profile of activation of autophagy, apoptosis and mTOR with respect to excessive level of ER stress. Our data further confirm the results published by Ogata et al. that autophagy always has a transient activation followed by apoptotic cell death (Fig 1B) [42]. Since no increase in cell death rate is observed during autophagy, these results indicate the protective role of autophagy in ER stress. The current observation also shows that mTOR has an interesting time profile when TG was added to the cells (see mTOR markers on Fig 1B). Although Kato et al. suggested that mTOR continuously gets activated during ER stress [25], our results show that mTOR has a transient inactivation. mTOR is always active at physiological conditions, which refers to its important role in maintaining cellular homeostasis. However its activity quickly drops when autophagy is active. This transient disappearance of mTOR seems logic since mTOR is a well-known negative regulator of autophagy [7]. For the effective autophagy-dependent survival the down-regulation of mTOR is crucial during ER stress. Later mTOR activity gets even stronger than at physiological condition corresponding to autophagy inactivation and apoptosis activation. Kato et al. has shown that mTOR is able to activate apoptotic cell death through UPR activation [25]. We extend this picture by following ER stress-dependent AMPK activity change as well. AMPK seems to be active during autophagic process; however its activity quickly drops when apoptosis turns on (Fig 1B). The importance of proper mTOR-AMPK balance during ER stress was also confirmed by addition of autophagy inhibitor (3MA) or mTOR inactivator (rapamycin) combined with TG. While 3MA+TG treatment diminished both autophagy-dependent survival and AMPK activity and hyper-activate mTOR pathway followed by early apoptotic cell death, addition of rapamycin+TG could maintain cell viability via massive autophagy (Fig 1C and 1D).

Previous results suggest that mTOR is down-regulated at various stress events (such as nutrient depletion, Huntingtin overexpression) *via* GADD34 [31–33]. Since GADD34 is one of the key components of ER-stress induced UPR, we investigated whether GADD34 has a negative effect on mTOR during ER stress. Using pharmacological inhibitor (guanabenz) or siRNA to block GADD34 our current observations show that GADD34 has a crucial effect to maintain the precise balance of life-and-death decision (Figs 2, 6A and 6B). GB was able to inhibit GADD34 resulting in a fast activation of both mTOR and apoptotic cell death, meanwhile autophagy-dependent survival was much shorter with respect to excessive level of ER stress. We also observed a quick decrease in cell viability (Fig 2). Similar effects were observed upon GADD34 silencing (Fig 6A and 6B). Here we show that GADD34 depletion resulted in a quick activation of mTOR markers suggesting that GADD34 is able to down-regulate apoptotic cell death *via* mTOR inhibition during ER stress.

We also demonstrate that the negative effect of GADD34 absence during ER stress (by GB treatment or using siGADD34) was suppressed by addition of mTOR inhibitor (Figs 3, 6A and 6B). Pre-treatment with rapamycin was able to postpone apoptotic cell death although GADD34 activity was missing. The rapamycin-dependent inhibition of mTOR did not let the speed up of apoptotic cell death. On the basis of the present findings we could extend our previous wiring diagram with a new component (Fig 6C); we suggest that GADD34, as one of the autophagy inducers helps autophagy-dependent survival *via* down-regulating mTOR during ER stress.

Parallel to UPR-induced GADD34, AMPK activity is also high during autophagy-dependent survival, but its activity quickly drops when apoptosis turns on during ER stress (Fig 1B). It is well-known that AMPK activation is essential for starvation-induced autophagy [43]; however, here we show that AMPK has some regulatory role with respect to ER stress, as well. Although many papers suggest that mTOR is regulated by GADD34 through TSC2 dephosphorylation [31, 44] and here we also confirm the connection between mTOR and GADD34, we cannot rule out that GADD34 has some direct or indirect effect on AMPK, too. As AMPK inhibits mTOR *via* various activators [45], it might be possible that GADD34 down-regulates mTOR through AMPK activation during ER stress. It is also possible that both direct (*via* TSC2) and indirect (*via* AMPK) regulatory connections are present between mTOR and GADD34 for the proper mTOR-AMPK balance with respect to ER stress. Further studies are needed to explore the regulatory connection between GADD34 and AMPK in details.

To further explain the importance of mTOR-AMPK balance in ER stress-dependent life-and-death decision, the phytochemicals resveratrol was used. Resveratrol was found to activate AMPK in HEK293 cells leading to autophagy [46]. Its negative effect on mTOR was also shown through Sirt1 activation [47]. Here we show that a 24 hours long pre-treatment with resveratrol followed by TG addition was able to extend cell viability *via* intensive presence of both AMPK and autophagy, meanwhile mTOR and apoptotic cell death were down-regulated (Fig 4). In this study we show resveratrol treatment mimics the effects of rapamycin; thus, the negative effect of GADD34 depletion (by GB or siGADD34) was successfully suppressed with both agents (Figs 5, 6A and 6B). These results further confirm that AMPK and mTOR pathways are highly connected to UPR *via* GADD34 with respect to ER stress.

Since ER stress is involved in various human pathologies such as neurodegenerative diseases, obesity, NASH, type two diabetes and many others, focusing on ER stress-induced life-and-death decision has medical importance. Further studies are needed to extrapolate the present findings to normal cells or to in vivo conditions. However, several observations might indicate that the mechanism described here is a general one. As an example, it has already proved that resveratrol were able to affect a wide range of cellular signal transduction pathways and it might have a therapeutic potential [48]. Resveratrol seems to be neuroprotective in several models of Huntingtin disease [49, 50]. Meanwhile Hyrskyluoto et al. has shown that overexpression of GADD34 were able to induce cytoprotective autophagy and down-regulate mTOR in mutant huntingtin expressing cells [32]. Here we demonstrated that GADD34 got hyper-activated in resveratrol treatment followed by addition of ER stressor suggesting that GADD34 might be one of the key elements of resveratrol-dependent neuroprotection in Huntingtin disease. Therefore our observations show that signalling pathways connecting ER stress to AMPK-mTOR imbalance and finally to life-and-death decision are potential druggable targets.

## Supporting Information

S1 Fig Time course profile of cell viability, autophagy and apoptosis in TG-induced ER stress. HEK293T cells were treated with TG (10 μM for two hours). **A)** The relative cell viability after TG treatment was denoted in time. **B)** Densitometry data represent the intensity of proCaspase-3, cleaved PARP, GADD34 normalised for GAPDH, LC3II normalized for LC3I, ULK-555P normalized for total level of ULK, 4-EBP1P normalized for total level of 4-EBP1 and p70S6-P normalized for total level of p70S6. Error bars represent standard deviation, asterisks indicate statistically significant difference from the control: ∗—p < 0.05; ∗∗—p < 0.01.
(TIF)

S2 Fig Time course profile of cell viability, autophagy and apoptosis in TG-induced ER stress with/without addition of autophagy inhibitor. HEK293T cells were pre-treated with 3-MA (1 mM for two hours) followed by TG addition (10 μM for two hours). **A)** The relative cell viability after TG treatment was denoted in time. **B)** Densitometry data represent the intensity of cleaved PARP, GADD34 normalised for GAPDH, LC3II normalized for LC3I, ULK-555P normalized for total level of ULK and 4-EBP1P normalized for total level of 4-EBP1. Error bars represent standard deviation, asterisks indicate statistically significant difference from the control: ∗—p < 0.05; ∗∗—p < 0.01.
(TIF)

S3 Fig Time course profile of cell viability, autophagy and apoptosis in TG-induced ER stress when autophagy was activated. HEK293T cells were pre-treated with rapamycin (100 nM for two hours) followed by TG addition (10 μM for two hours). **A)** The relative cell viability after TG treatment was denoted in time. **B)** Densitometry data represent the intensity of cleaved PARP, GADD34 normalised for GAPDH, LC3II normalized for LC3I, ULK-555P normalized for total level of ULK and 4-EBP1P normalized for total level of 4-EBP1. Error bars represent standard deviation, asterisks indicate statistically significant difference from the control: ∗—p < 0.05; ∗∗—p < 0.01.
(TIF)

S4 Fig Analysing autophagy activation in the presence of an autophagic flux inhibitor. HEK293T cells were pre-treated without/with Bafilomycin A (100 nM Baf for two hours) followed by rapamycin (100 nM for two hours), 3-MA (1 mM for two hours) or TG (10 μM for 30 mins) addition. The Rap and 3-MA treatment was combined with TG (10 μM for 30 mins). **A)** The relative number of viable cells after TG treatment was denoted in time. **B)** The autophagy (LC3, p63) and the apoptosis (PARP, proCaspase-3) markers were followed in time by immunoblotting. GAPDH was used as loading control. **C)** Densitometry data represent the intensity of proCaspase-3, cleaved PARP, p62 normalised for GAPDH and LC3II normalized for LC3I. Error bars represent standard deviation, asterisks indicate statistically significant difference from the control: ∗—p < 0.05; ∗∗—p < 0.01.
(TIF)

S5 Fig The effect of the GADD34 inhibitor guanabenz (GB) on cell viability in TG-induced ER stress. HEK293T cells were treated with various concentration of GB for one hour. The relative cell viability after GB treatment was denoted (error bars represent standard deviation, asterisks indicate statistically significant difference from the control: ∗—p < 0.05; ∗∗—p < 0.01).
(TIF)

S6 Fig Time course profile of cell viability, autophagy and apoptosis in TG-induced ER stress when GADD34 was inhibited. HEK293T cells were pre-treated with GB (5 μM for one hour) followed by TG addition (10 μM for two hours). The GB level was kept high until end of the cell treatment. **A)** The relative cell viability after TG treatment was denoted in time. **B)** Densitometry data represent the intensity of cleaved PARP normalised for GAPDH, LC3II normalized for LC3I, eiF2α-P normalized for total level of eiF2α, ULK-555P normalized for total level of ULK and 4-EBP1P normalized for total level of 4-EBP1. Error bars represent standard deviation, asterisks indicate statistically significant difference from the control: ∗—p < 0.05; ∗∗—p < 0.01.
(TIF)

S7 Fig The effect of GADD34 inhibition with respect to ER stress using another cell line. HepG2 cells were pre-treated with GB (5 μM for one hour) followed by TG addition (25 μM for two hours). The GB level was kept high until end of the cell treatment. **A)** The relative number of viable cell was denoted in time after TG treatment. **B)** The autophagy (LC3), the apoptosis (proCaspase-3), the AMPK (ULK-555P) and the mTOR (4-EBP1P) markers and eiF2αP were followed in time by immunoblotting. GAPDH was used as loading control. **C)** Densitometry data represent the intensity of proCaspase-3 normalised for GAPDH, LC3II normalized for LC3I, eiF2α-P normalized for total level of eiF2α, ULK-555P normalized for total level of ULK and 4-EBP1P normalized for total level of 4-EBP1. Error bars represent standard deviation, asterisks indicate statistically significant difference from the control: ∗—p < 0.05; ∗∗—p < 0.01.
(TIF)

S8 Fig The effect of GADD34 inhibition with respect to ER stress using another ER stressor. HEK293T cells were pre-treated with GB (5 μM for one hour) followed by TM addition (100 μM for two hours). The GB level was kept high until end of the cell treatment. **A)** The relative number of viable cell was denoted in time after TM treatment. **B)** The autophagy (LC3), the apoptosis (PARP), the AMPK (ULK-555P) and the mTOR (4-EBP1P) markers and eiF2αP were followed in time by immunoblotting. GAPDH was used as loading control. **C)** Densitometry data represent the intensity of cleaved PARP normalised for GAPDH, LC3II normalized for LC3I, eiF2α-P normalized for total level of eiF2α, ULK-555P normalized for total level of ULK and 4-EBP1P normalized for total level of 4-EBP1. Error bars represent standard deviation, asterisks indicate statistically significant difference from the control: ∗—p < 0.05; ∗∗—p < 0.01.
(TIF)

S9 Fig Time course profile of cell viability, autophagy and apoptosis in TG-induced ER stress when both GADD34 and mTOR was inhibited. HEK293T cells were pre-treated with GB (5 μM for one hour) then with rapamycin (100 nM for two hours) followed by TG addition (10 μM for two hours). The GB level was kept high until end of the cell treatment. **A)** The relative cell viability after TG treatment was denoted in time. **B)** Densitometry data represent the intensity of cleaved PARP normalised for GAPDH, LC3II normalized for LC3I, eiF2α-P normalized for total level of eiF2α, ULK-555P normalized for total level of ULK and 4-EBP1P normalized for total level of 4-EBP1. Error bars represent standard deviation, asterisks indicate statistically significant difference from the control: ∗—p < 0.05; ∗∗—p < 0.01.
(TIF)

S10 Fig Time course profile of cell viability, autophagy and apoptosis in TG-induced ER stress when autophagy was hyper-activated by resveratrol. HEK293T cells were pre-treated with resveratrol (10 μM for twenty-four hours) followed by TG addition (10 μM for two hours). **A)** The relative cell viability after TG treatment was denoted in time. **B)** Densitometry data represent the intensity of proCaspase-3, cleaved PARP, GADD34 normalised for GAPDH, LC3II normalized for LC3I, ULK-555P normalized for total level of ULK, 4-EBP1P normalized for total level of 4-EBP1 and p70S6-P normalized for total level of p70S6. Error bars represent standard deviation, asterisks indicate statistically significant difference from the control: ∗—p < 0.05; ∗∗—p < 0.01.
(TIF)

S11 Fig Time course profile of cell viability, autophagy and apoptosis in TG-induced ER stress when GADD34 inhibition is combined with resveratrol addition. HEK293T cells were pre-treated with GB (5 μM for one hours) then with resveratrol (10 μM for twenty-four hours) followed by TG addition (10 μM for two hours). The GB level was kept high until end of the cell treatment. **A)** The relative cell viability after TG treatment was denoted in time. **B)** Densitometry data represent the intensity of cleaved PARP normalised for GAPDH, LC3II normalized for LC3I, eiF2α-P normalized for total level of eiF2α, ULK-555P normalized for total level of ULK and 4-EBP1P normalized for total level of 4-EBP1. Error bars represent standard deviation, asterisks indicate statistically significant difference from the control: ∗—p < 0.05; ∗∗—p < 0.01.
(TIF)

S12 Fig Testing the effect of GADD34 silencing with respect to ER stress. GADD34 was silenced in HEK293T cells, then cells were treated with 10 μM TG for two hours and pre-treated with resveratrol (10 μM for twenty-four hours) followed by TG addition (10 μM for two hours). The successful GADD34 silencing was demonstrated both by **A)** real-time PCR and **B)** Western blot analysis. **C)** Densitometry data represent the intensity of cleaved PARP normalised for GAPDH, LC3II normalized for LC3I, eiF2α-P normalized for total level of eiF2α, ULK-555P normalized for total level of ULK and 4-EBP1P normalized for total level of 4-EBP1. Error bars represent standard deviation, asterisks indicate statistically significant difference from the control: ∗—p < 0.05; ∗∗—p < 0.01.
(TIF)
