# Supplementary figures and images for: GADD34 Keeps the mTOR Pathway Inactivated in Endoplasmic Reticulum Stress Related Autophagy
Source: PLoS One. 2016 Dec 16;11(12):e0168359. doi: 10.1371/journal.pone.0168359 (PMC5161374; doi:10.1371/journal.pone.0168359)

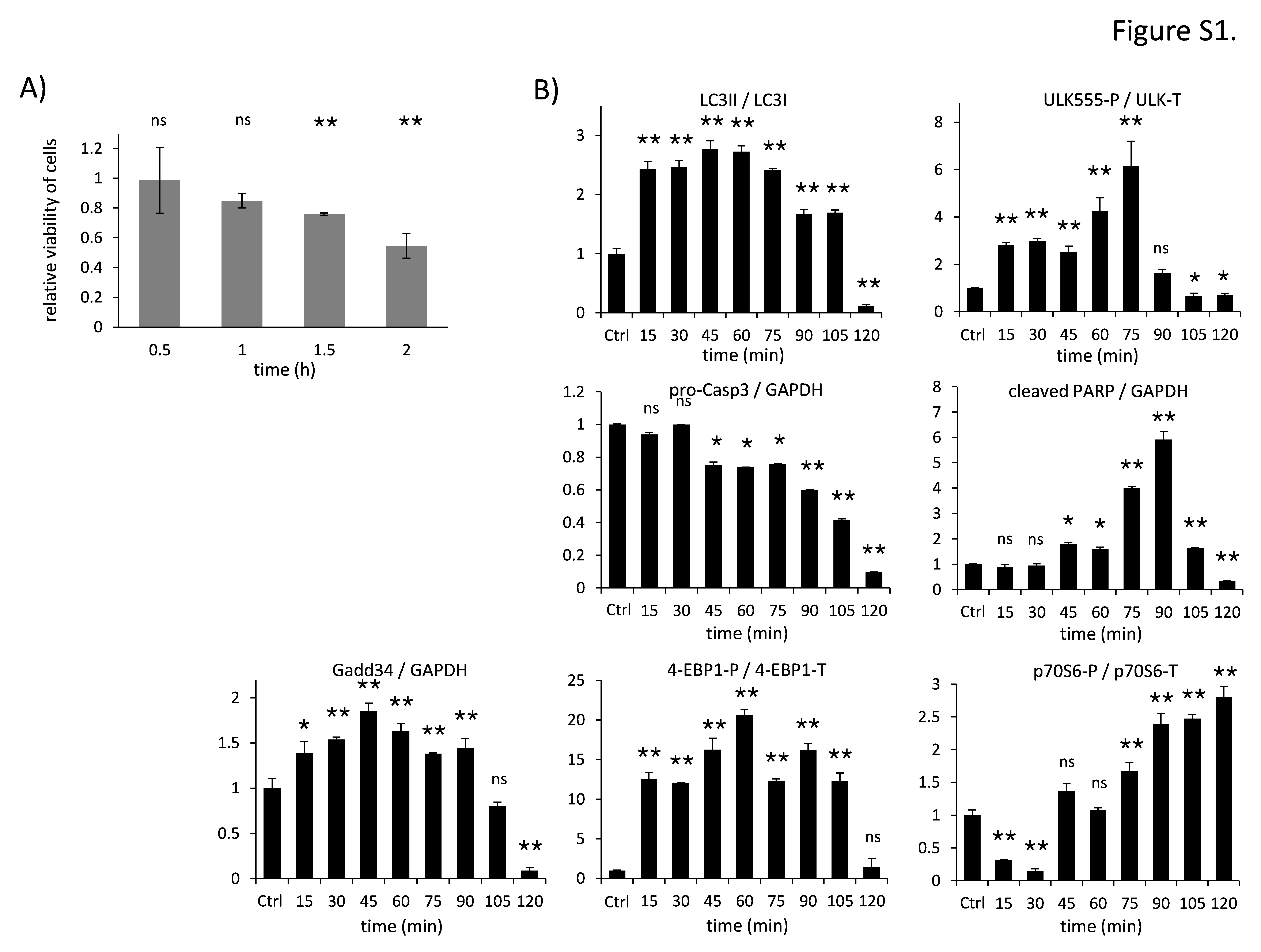

Supplement: S1 Fig — HEK293T cells were treated with TG (10 μM for two hours). A) The relative cell viability after TG treatment was denoted in time. B) Densitometry data represent the intensity of proCaspase-3, cleaved PARP, GADD34 normalised for GAPDH, LC3II normalized for LC3I, ULK-555P normalized for total level of ULK, 4-EBP1P normalized for total level of 4-EBP1 and p70S6-P normalized for total level of p70S6. Error bars represent standard deviation, asterisks indicate statistically significant difference from the control: ∗—p < 0.05; ∗∗—p < 0.01. (TIF) [file pone.0168359.s001.tif]

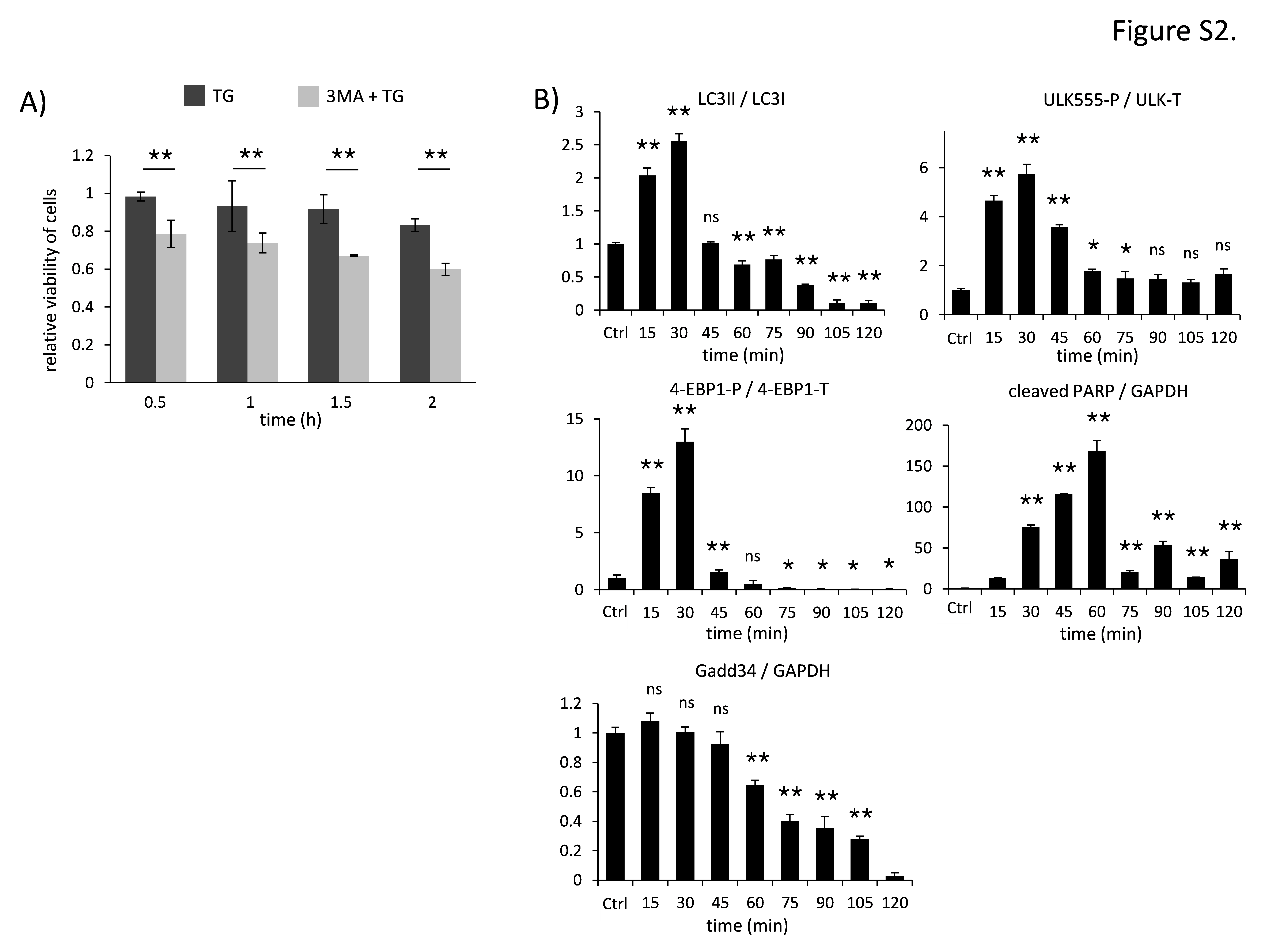

Supplement: S2 Fig — HEK293T cells were pre-treated with 3-MA (1 mM for two hours) followed by TG addition (10 μM for two hours). A) The relative cell viability after TG treatment was denoted in time. B) Densitometry data represent the intensity of cleaved PARP, GADD34 normalised for GAPDH, LC3II normalized for LC3I, ULK-555P normalized for total level of ULK and 4-EBP1P normalized for total level of 4-EBP1. Error bars represent standard deviation, asterisks indicate statistically significant difference from the control: ∗—p < 0.05; ∗∗—p < 0.01. (TIF) [file pone.0168359.s002.tif]

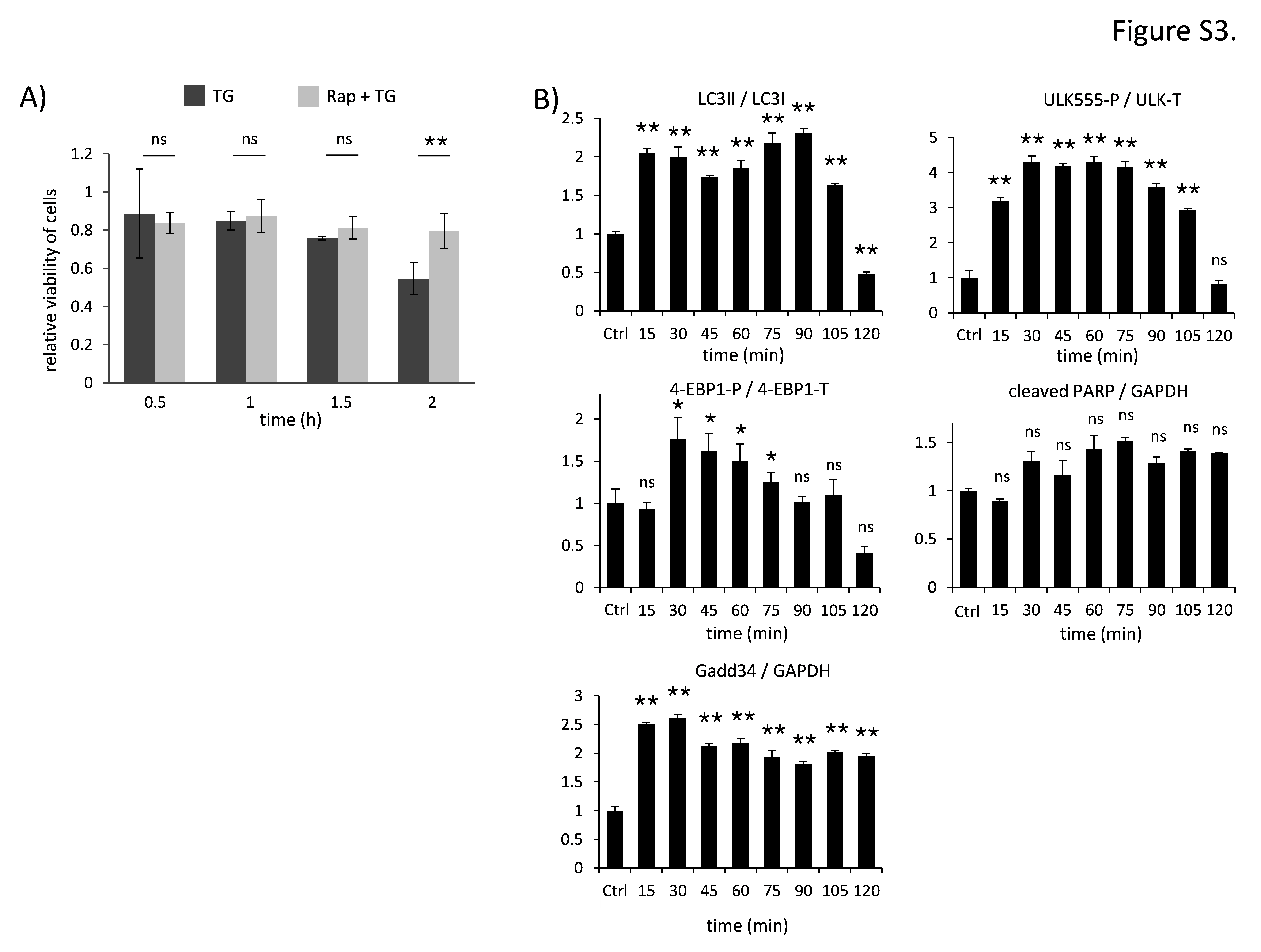

Supplement: S3 Fig — HEK293T cells were pre-treated with rapamycin (100 nM for two hours) followed by TG addition (10 μM for two hours). A) The relative cell viability after TG treatment was denoted in time. B) Densitometry data represent the intensity of cleaved PARP, GADD34 normalised for GAPDH, LC3II normalized for LC3I, ULK-555P normalized for total level of ULK and 4-EBP1P normalized for total level of 4-EBP1. Error bars represent standard deviation, asterisks indicate statistically significant difference from the control: ∗—p < 0.05; ∗∗—p < 0.01. (TIF) [file pone.0168359.s003.tif]

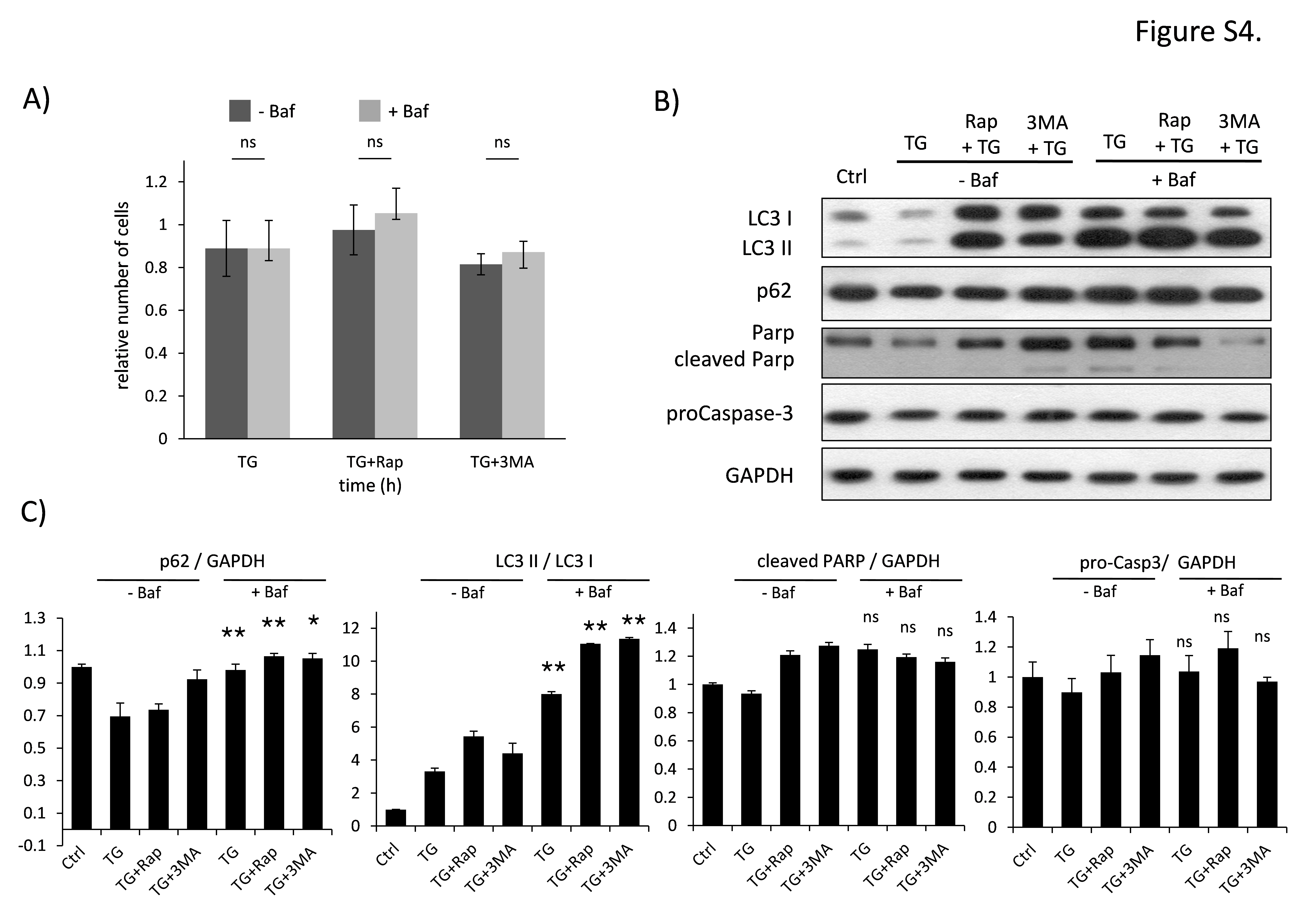

Supplement: S4 Fig — HEK293T cells were pre-treated without/with Bafilomycin A (100 nM Baf for two hours) followed by rapamycin (100 nM for two hours), 3-MA (1 mM for two hours) or TG (10 μM for 30 mins) addition. The Rap and 3-MA treatment was combined with TG (10 μM for 30 mins). A) The relative number of viable cells after TG treatment was denoted in time. B) The autophagy (LC3, p63) and the apoptosis (PARP, proCaspase-3) markers were followed in time by immunoblotting. GAPDH was used as loading control. C) Densitometry data represent the intensity of proCaspase-3, cleaved PARP, p62 normalised for GAPDH and LC3II normalized for LC3I. Error bars represent standard deviation, asterisks indicate statistically significant difference from the control: ∗—p < 0.05; ∗∗—p < 0.01. (TIF) [file pone.0168359.s004.tif]

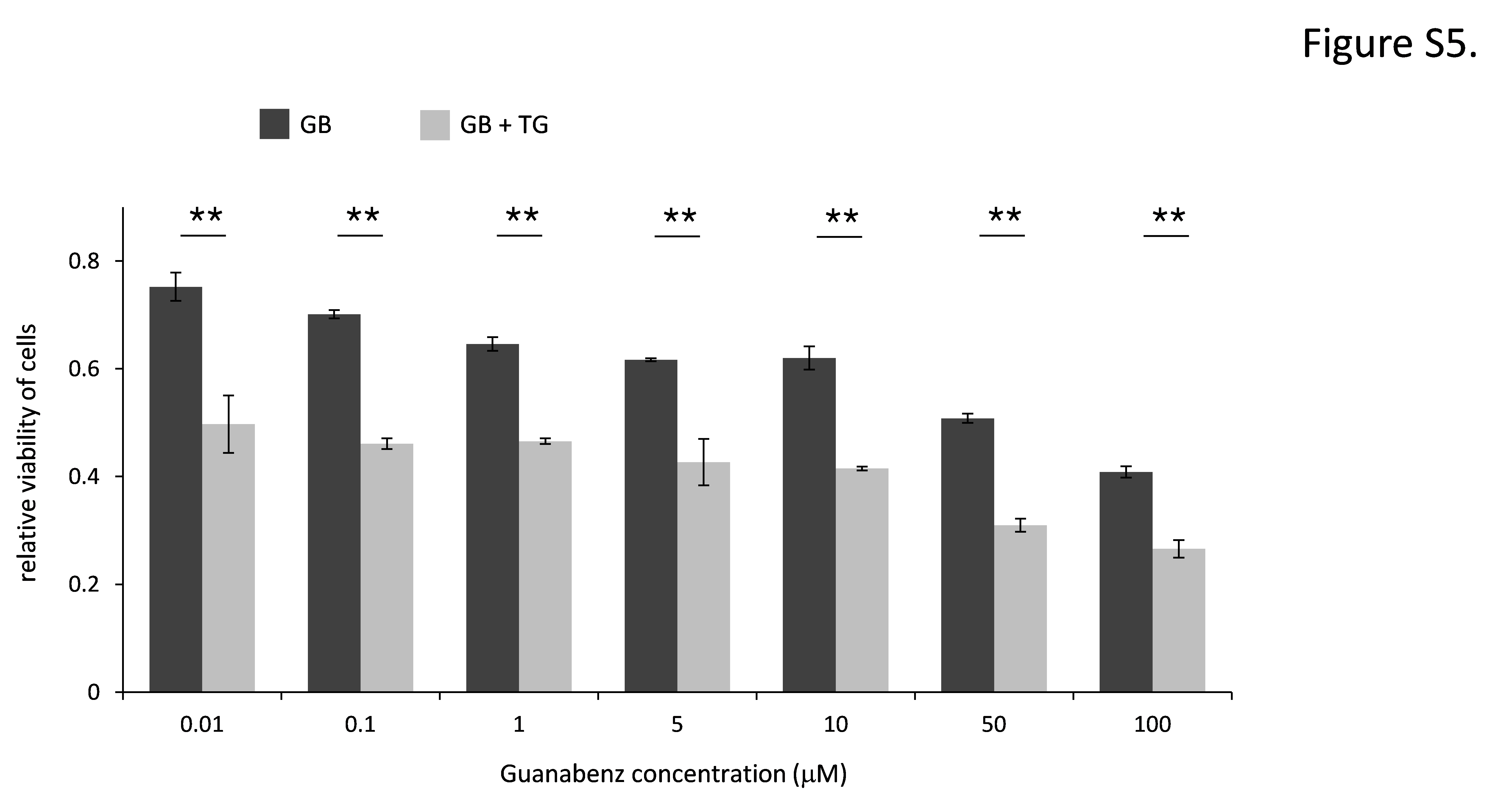

Supplement: S5 Fig — HEK293T cells were treated with various concentration of GB for one hour. The relative cell viability after GB treatment was denoted (error bars represent standard deviation, asterisks indicate statistically significant difference from the control: ∗—p < 0.05; ∗∗—p < 0.01). (TIF) [file pone.0168359.s005.tif]

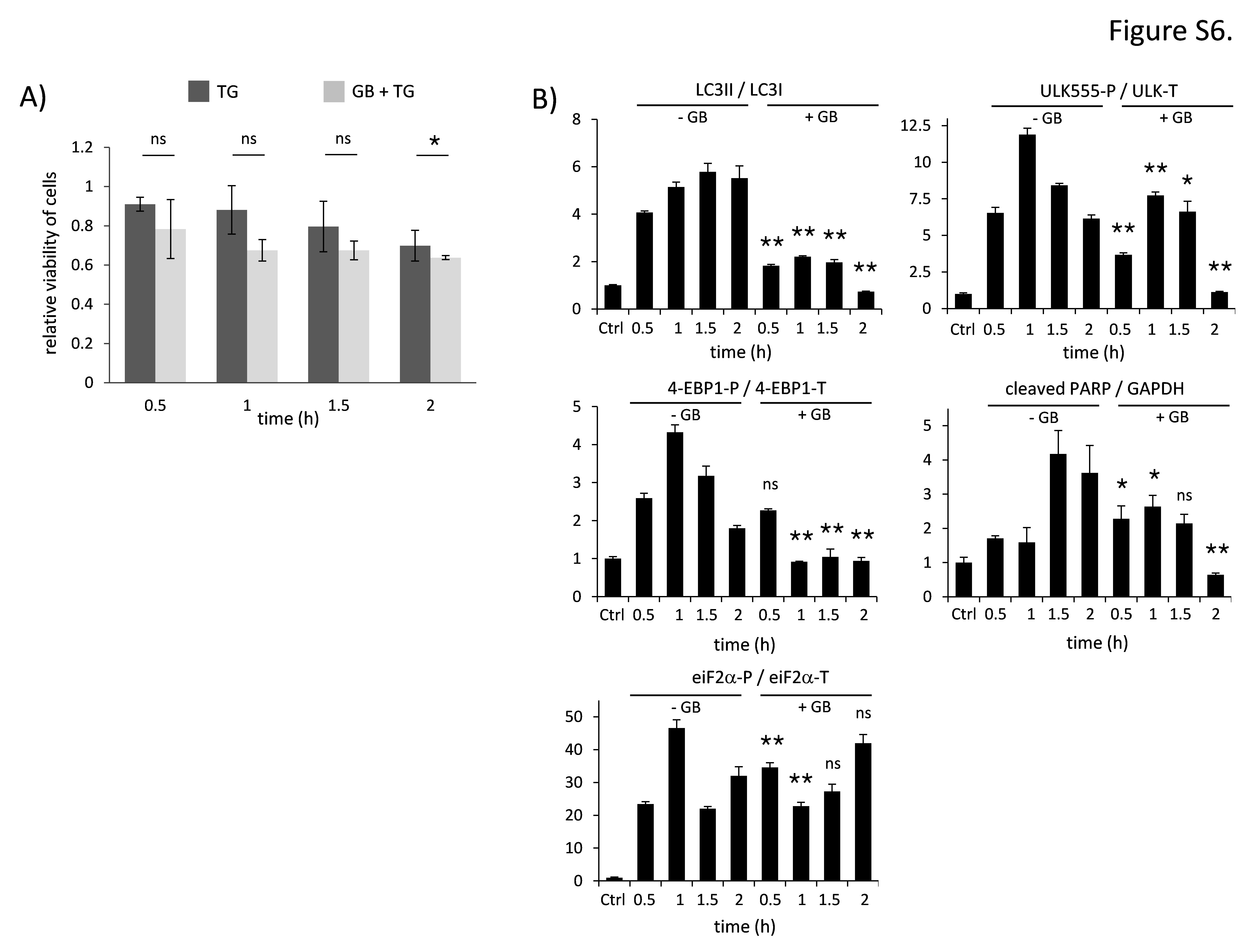

Supplement: S6 Fig — HEK293T cells were pre-treated with GB (5 μM for one hour) followed by TG addition (10 μM for two hours). The GB level was kept high until end of the cell treatment. A) The relative cell viability after TG treatment was denoted in time. B) Densitometry data represent the intensity of cleaved PARP normalised for GAPDH, LC3II normalized for LC3I, eiF2α-P normalized for total level of eiF2α, ULK-555P normalized for total level of ULK and 4-EBP1P normalized for total level of 4-EBP1. Error bars represent standard deviation, asterisks indicate statistically significant difference from the control: ∗—p < 0.05; ∗∗—p < 0.01. (TIF) [file pone.0168359.s006.tif]

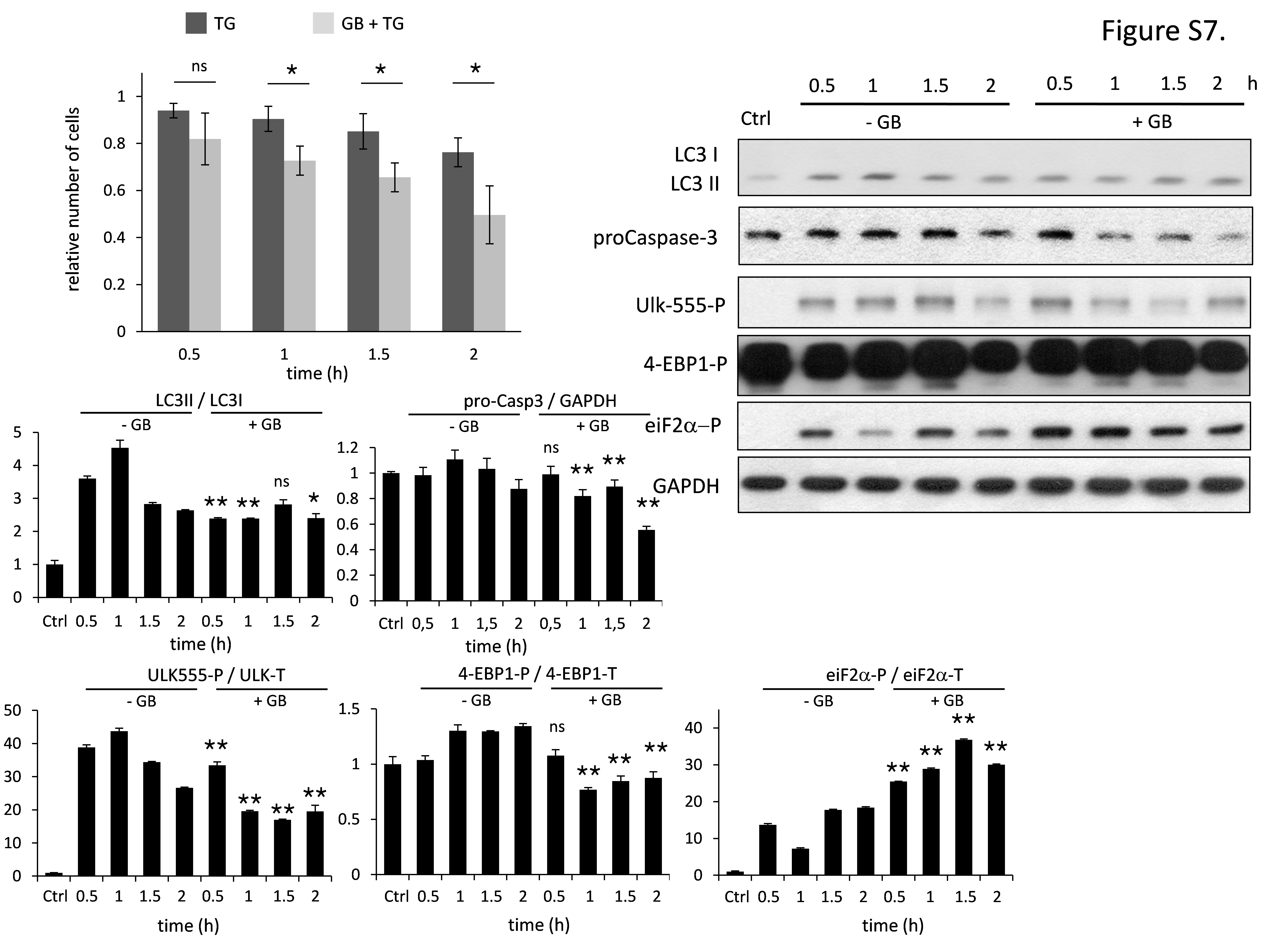

Supplement: S7 Fig — HepG2 cells were pre-treated with GB (5 μM for one hour) followed by TG addition (25 μM for two hours). The GB level was kept high until end of the cell treatment. A) The relative number of viable cell was denoted in time after TG treatment. B) The autophagy (LC3), the apoptosis (proCaspase-3), the AMPK (ULK-555P) and the mTOR (4-EBP1P) markers and eiF2αP were followed in time by immunoblotting. GAPDH was used as loading control. C) Densitometry data represent the intensity of proCaspase-3 normalised for GAPDH, LC3II normalized for LC3I, eiF2α-P normalized for total level of eiF2α, ULK-555P normalized for total level of ULK and 4-EBP1P normalized for total level of 4-EBP1. Error bars represent standard deviation, asterisks indicate statistically significant difference from the control: ∗—p < 0.05; ∗∗—p < 0.01. (TIF) [file pone.0168359.s007.tif]

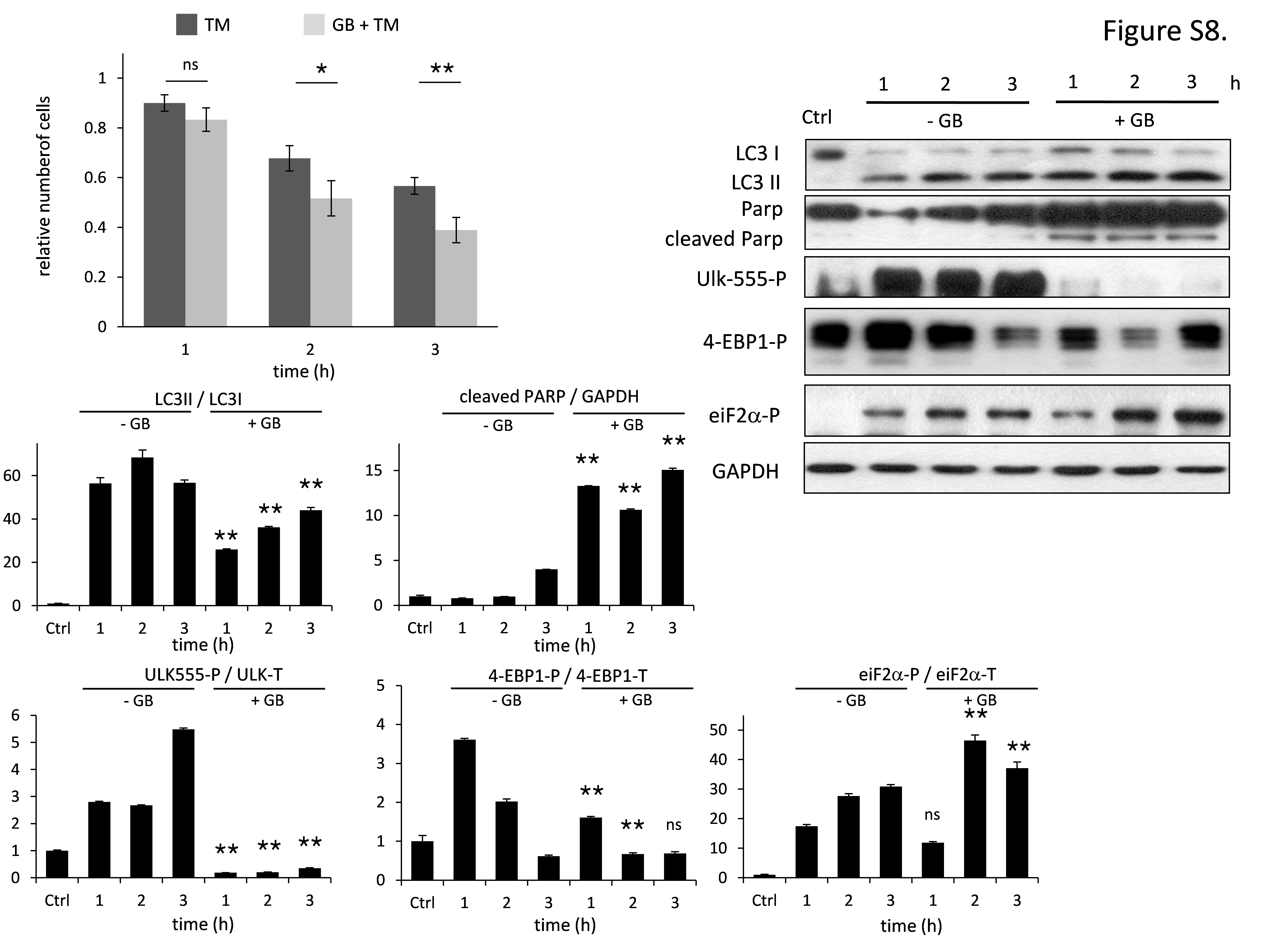

Supplement: S8 Fig — HEK293T cells were pre-treated with GB (5 μM for one hour) followed by TM addition (100 μM for two hours). The GB level was kept high until end of the cell treatment. A) The relative number of viable cell was denoted in time after TM treatment. B) The autophagy (LC3), the apoptosis (PARP), the AMPK (ULK-555P) and the mTOR (4-EBP1P) markers and eiF2αP were followed in time by immunoblotting. GAPDH was used as loading control. C) Densitometry data represent the intensity of cleaved PARP normalised for GAPDH, LC3II normalized for LC3I, eiF2α-P normalized for total level of eiF2α, ULK-555P normalized for total level of ULK and 4-EBP1P normalized for total level of 4-EBP1. Error bars represent standard deviation, asterisks indicate statistically significant difference from the control: ∗—p < 0.05; ∗∗—p < 0.01. (TIF) [file pone.0168359.s008.tif]

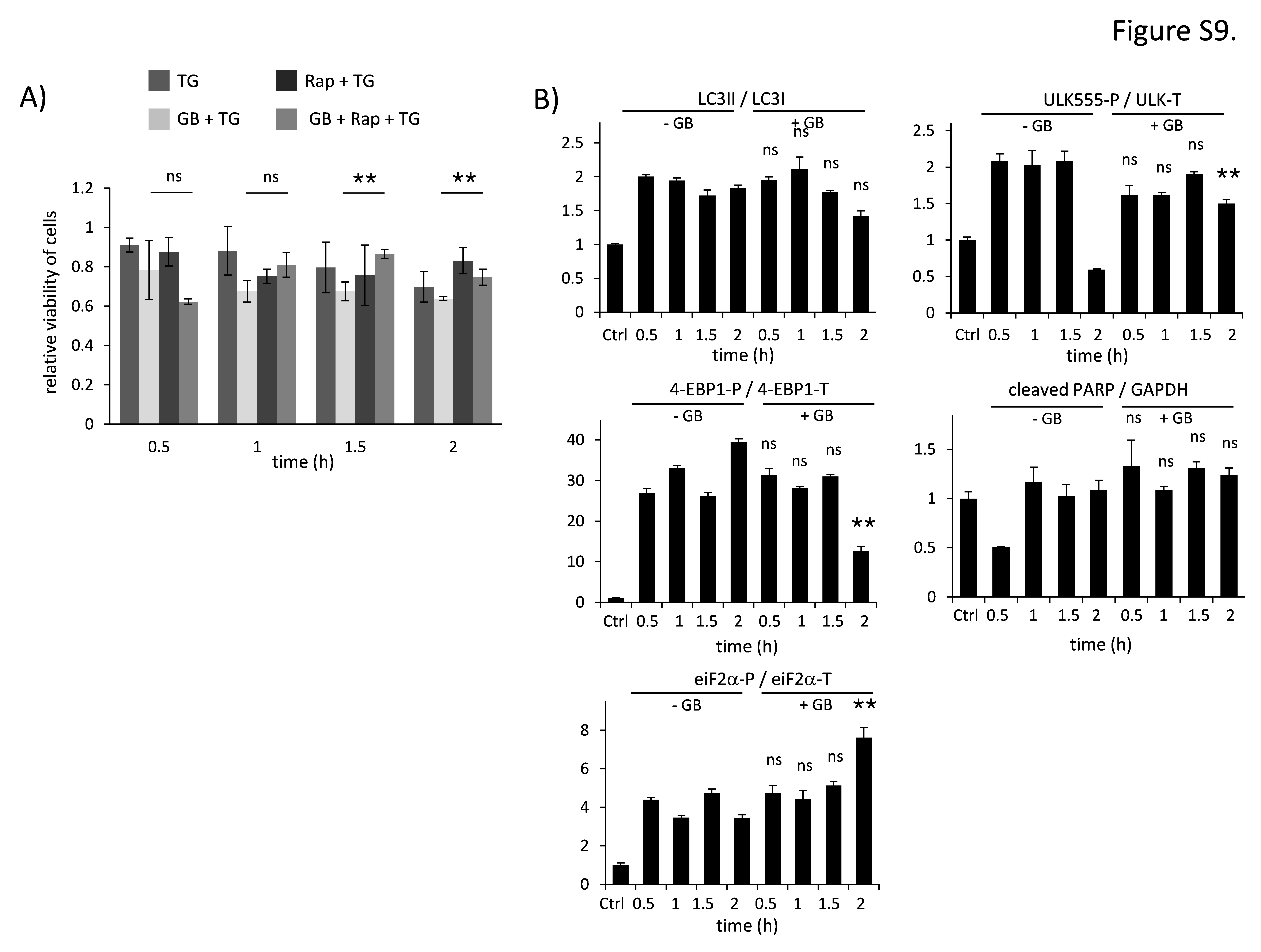

Supplement: S9 Fig — HEK293T cells were pre-treated with GB (5 μM for one hour) then with rapamycin (100 nM for two hours) followed by TG addition (10 μM for two hours). The GB level was kept high until end of the cell treatment. A) The relative cell viability after TG treatment was denoted in time. B) Densitometry data represent the intensity of cleaved PARP normalised for GAPDH, LC3II normalized for LC3I, eiF2α-P normalized for total level of eiF2α, ULK-555P normalized for total level of ULK and 4-EBP1P normalized for total level of 4-EBP1. Error bars represent standard deviation, asterisks indicate statistically significant difference from the control: ∗—p < 0.05; ∗∗—p < 0.01. (TIF) [file pone.0168359.s009.tif]

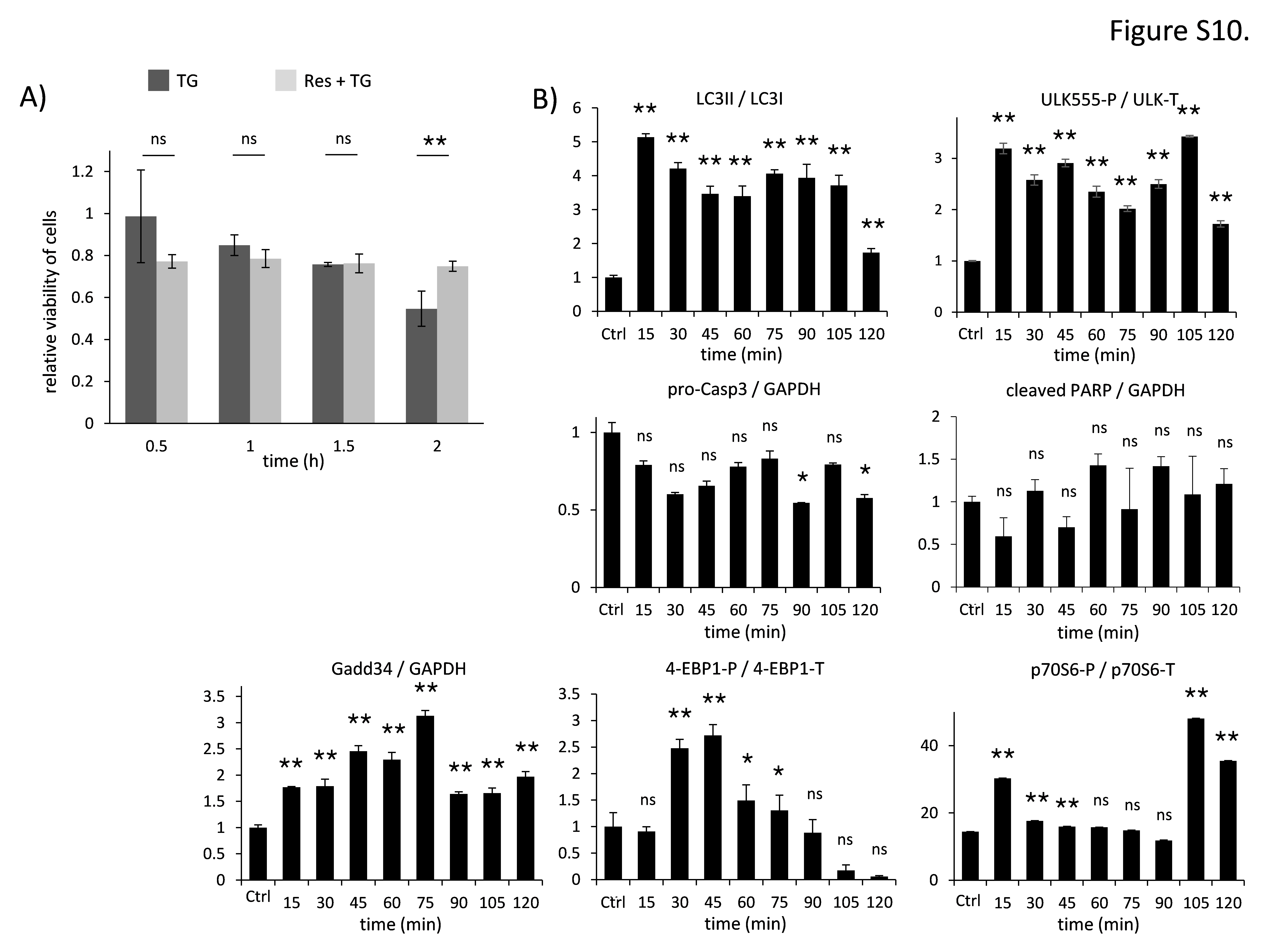

Supplement: S10 Fig — HEK293T cells were pre-treated with resveratrol (10 μM for twenty-four hours) followed by TG addition (10 μM for two hours). A) The relative cell viability after TG treatment was denoted in time. B) Densitometry data represent the intensity of proCaspase-3, cleaved PARP, GADD34 normalised for GAPDH, LC3II normalized for LC3I, ULK-555P normalized for total level of ULK, 4-EBP1P normalized for total level of 4-EBP1 and p70S6-P normalized for total level of p70S6. Error bars represent standard deviation, asterisks indicate statistically significant difference from the control: ∗—p < 0.05; ∗∗—p < 0.01. (TIF) [file pone.0168359.s010.tif]

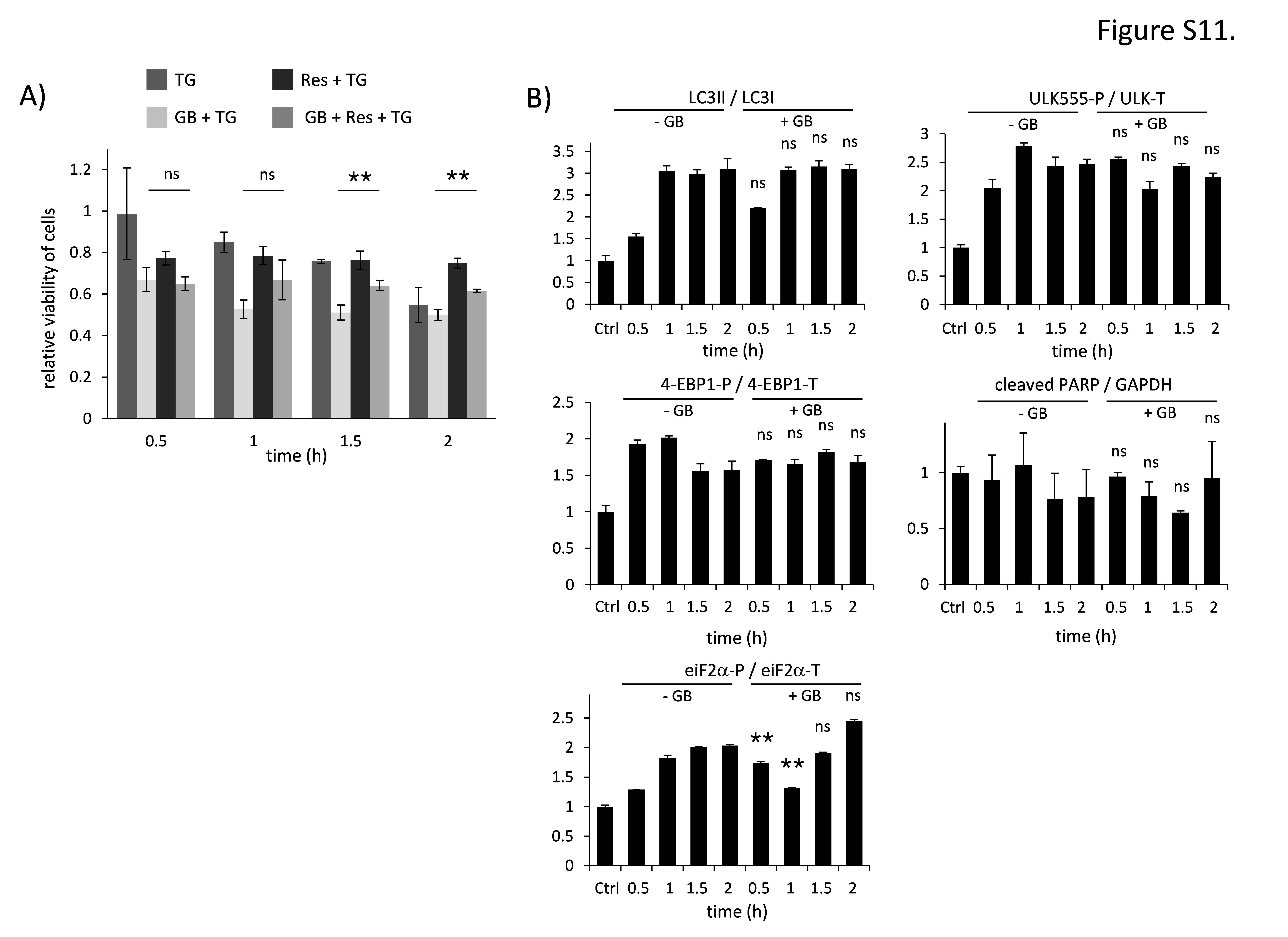

Supplement: S11 Fig — HEK293T cells were pre-treated with GB (5 μM for one hours) then with resveratrol (10 μM for twenty-four hours) followed by TG addition (10 μM for two hours). The GB level was kept high until end of the cell treatment. A) The relative cell viability after TG treatment was denoted in time. B) Densitometry data represent the intensity of cleaved PARP normalised for GAPDH, LC3II normalized for LC3I, eiF2α-P normalized for total level of eiF2α, ULK-555P normalized for total level of ULK and 4-EBP1P normalized for total level of 4-EBP1. Error bars represent standard deviation, asterisks indicate statistically significant difference from the control: ∗—p < 0.05; ∗∗—p < 0.01. (TIF) [file pone.0168359.s011.tif]

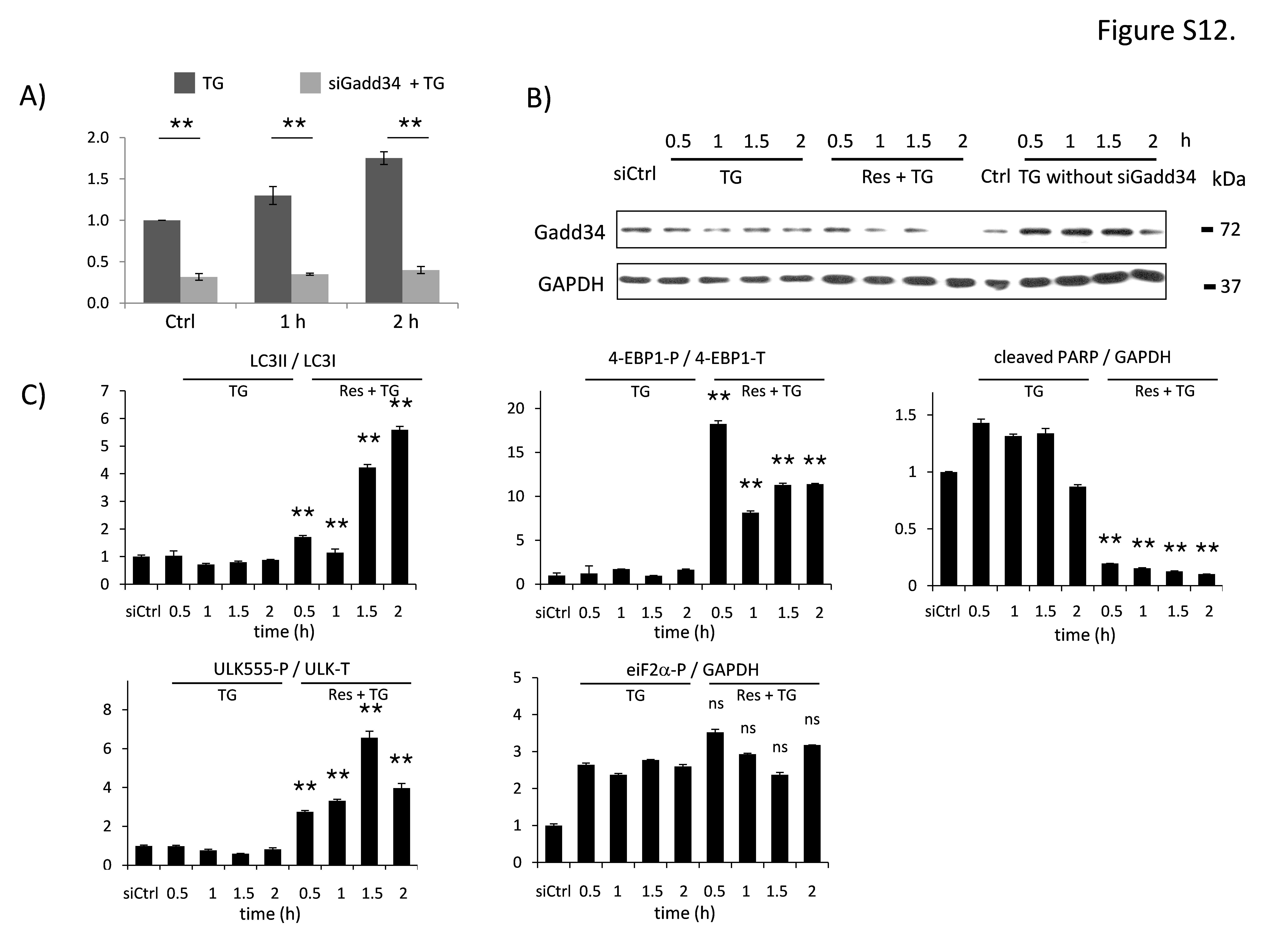

Supplement: S12 Fig — GADD34 was silenced in HEK293T cells, then cells were treated with 10 μM TG for two hours and pre-treated with resveratrol (10 μM for twenty-four hours) followed by TG addition (10 μM for two hours). The successful GADD34 silencing was demonstrated both by A) real-time PCR and B) Western blot analysis. C) Densitometry data represent the intensity of cleaved PARP normalised for GAPDH, LC3II normalized for LC3I, eiF2α-P normalized for total level of eiF2α, ULK-555P normalized for total level of ULK and 4-EBP1P normalized for total level of 4-EBP1. Error bars represent standard deviation, asterisks indicate statistically significant difference from the control: ∗—p < 0.05; ∗∗—p < 0.01. (TIF) [file pone.0168359.s012.tif]
